# Human brain network for reading the mind from the eyes: beyond activated and deactivated regions

**DOI:** 10.1093/scan/nsag042

**Published:** 2026-06-03

**Authors:** Maya Zheltyakova, Maxim Kireev, Artem Myznikov, Ruslan Masharipov, Mikhail Didur, Denis Cherednichenko, Alexander Korotkov

**Affiliations:** N.P. Bechtereva Institute of the Human Brain, Russian Academy of Science, Saint Petersburg, Russia; N.P. Bechtereva Institute of the Human Brain, Russian Academy of Science, Saint Petersburg, Russia; Institute for Cognitive Studies, Saint Petersburg State University, Saint Petersburg, Russia; N.P. Bechtereva Institute of the Human Brain, Russian Academy of Science, Saint Petersburg, Russia; N.P. Bechtereva Institute of the Human Brain, Russian Academy of Science, Saint Petersburg, Russia; N.P. Bechtereva Institute of the Human Brain, Russian Academy of Science, Saint Petersburg, Russia; N.P. Bechtereva Institute of the Human Brain, Russian Academy of Science, Saint Petersburg, Russia; N.P. Bechtereva Institute of the Human Brain, Russian Academy of Science, Saint Petersburg, Russia

**Keywords:** BOLD signal, Theory of Mind, Reading the Mind in the Eyes Test, RMET, 27 temporoparietal junction

## Abstract

BOLD signal changes in the Theory of Mind (ToM) network often reflect reduced deactivation during target conditions, yet these below-baseline effects remain largely unaddressed. Using fMRI data from an extended version of the Reading the Mind in the Eyes Test, we examined task-modulated functional connectivity (TMFC) of these areas. We revealed that, despite below-baseline BOLD responses during the reading the mind in the eyes (RME) task, TPJ-pSTS subregions in the left middle and superior temporal gyri, and angular gyrus (AG) demonstrated TMFC patterns closely resembling those of ToM network areas in the pSTS and inferior frontal gyrus (IFG) showing above-baseline activations. The RME task-associated network included regions supporting affective mentalizing (bilateral TPJ-pSTS, IFG, middle frontal gyri, precentral gyri, supplementary motor area) and face-processing areas. These areas also elicited reduced TMFC with ToM network subregions in the AG, supramarginal gyrus, precuneus, and medial prefrontal cortex, associated with cognitive mentalizing. These findings demonstrate that regions with greater BOLD responses during the RME task, despite these responses remaining below baseline, are functionally engaged. Together, the results indicate a dynamic interaction between ToM network subcomponents during the RME task, characterized by engagement of the affective subcomponent and its functional segregation from the cognitive subcomponent.

## Introduction

The neural basis of human social interaction includes specialized brain regions associated with the ability to understand and predict the behavior of other people based on their internal mental states, which are not directly observable, including desires, beliefs, intentions, etc. (Adolphs 2009). The corresponding neural network is called the Theory of Mind (ToM) or the mentalizing network, and advances in this field have largely been enabled by neuroimaging techniques such as functional magnetic resonance imaging (fMRI) (Frith and Frith 2021). One important distinction has been proposed: depending on whether the inferred mental states have an emotional (affective) component or not, the corresponding neural structures can be divided into affective and cognitive ToM subcomponents (Shamay-Tsoory *et al.* 2007, Bodden *et al.* 2013).

Specifically, meta-analytic studies have identified the medial prefrontal cortex (mPFC), bilateral temporoparietal junction/posterior superior temporal sulcus (TPJ/pSTS), and precuneus/posterior cingulate cortex (precuneus/PCC) as key nodes of the ToM network, which are engaged in mentalizing across a wide range of tasks and stimulus types (Van Overwalle 2009, Mar 2011, Bzdok *et al.* 2012, Schurz *et al.* 2014, Molenberghs *et al.* 2016, Arioli *et al.* 2021, Diveica *et al.* 2021, Schurz *et al.* 2021, Balgova *et al.* 2024, Van Overwalle *et al.* 2026). At the same time, affective (“hot”) ToM tasks have been shown to rely on a broader network than that typically associated with more cognitive (“cold”) aspects of mentalizing. Meta-analytic evidence indicates greater activation during affective ToM tasks in regions including the left orbitofrontal cortex (OFC), bilateral pars opercularis and adjacent ventral premotor cortex (Molenberghs *et al.* 2016), as well as the left superior and middle temporal pole, supplementary motor area (SMA), and inferior frontal gyrus (IFG) (Arioli *et al.* 2021).

Although in fMRI studies the inclusion of a brain area in the ToM network and its specific subcomponent is typically determined based on increased local BOLD signal during an experimental condition relative to a control condition, this approach may overlook other important aspects of neural processing.

### BOLD signal’s change versus baseline in ToM network brain areas

As early as in/by 2004, researchers emphasized the importance of comparing task-induced neural activity not only across conditions but also relative to baseline, particularly in socially relevant paradigms aimed at studying brain mechanisms of social interactions (Iacoboni *et al.* 2004). Discussing mPFC involvement, Iacoboni *et al.* noted that so-called “activations” in social cognition tasks often reflect less deactivation during the target condition than during the control condition, rather than an absolute increase in neural activity. Despite this insight, only a few neuroimaging studies explicitly report the directionality of BOLD signal changes when describing the involvement of brain mechanisms of social interactions (Mitchell et al. 2002, Saxe and Kanwisher 2003, Elliott *et al.* 2006, Harris *et al.* 2007, Mitchell 2008, Cheng *et al.* 2010, Dodell-Feder *et al.* 2011, Barman *et al.* 2015, Alkire *et al.* 2018, Bitsch *et al.* 2018, Shain *et al.* 2023). Whenever deactivations were mentioned in the literature, they were described as “atypical” or “unclear” and remained largely unaddressed in terms of theoretical interpretation, as described in more detail below. Accordingly, some prior works have demonstrated brain deactivations under social conditions, though often without in-depth interpretation. For example, Mitchell *et al*. (2002) observed minimal deactivation in the mPFC, superior temporal cortex, intraparietal sulcus, and fusiform gyrus during person-judgments (e.g. matching names to personal traits), whereas significant deactivations were seen during object-related judgments. The authors discussed baseline processes and compared them to person judgement tasks but did not explore the meaning of the observed deactivations. Similarly, Mitchell (2008) found that reading stories about false beliefs (a variant of standard ToM task) elicited greater responses in the right TPJ, mPFC, and precuneus compared to control stories about outdated physical representations (e.g. maps or photos). However, both conditions produced BOLD signal decreases relative to baseline in the right TPJ, raising questions about how such results should be interpreted. The authors suggested that these effects could be induced by inconsistencies across laboratories stemming from differences in statistical modeling, but the functional implications of these deactivations remained unaddressed. In another study applying a standard ToM task, BOLD signals in the mPFC were decreased below baseline in both conditions but during reading stories about false beliefs the level of deactivation was relatively smaller compared to control stories (Dodell-Feder *et al.* 2011). However, the authors only discussed relative differences between conditions without comparing them to the baseline level of activity. Saxe and Kanwisher (2003) reported similar results in the precuneus: it was deactivated below baseline during both ToM and mechanical inference stories, but less so during ToM. According to the authors, it remained unclear whether the observed difference represented a true ToM-related response or simply a differential suppression effect, and the precuneus was excluded from further analysis. These findings highlight that alterations in the BOLD signal’s direction, in addition to its magnitude, could enhance our comprehension of the brain’s processing of social information, though this phenomenon has been largely understudied.

Filling in this gap, we analyzed not only relative BOLD signal changes but also their direction relative to baseline associated with widely used research tool designed to assess affective mentalization–the Reading the Mind in the Eyes Test (RMET).

### RMET application and limitations

Originally developed by Baron-Cohen *et al*. (1997; updated 2001) (Baron-Cohen *et al.* 1999, 2001), the test consists of 36 photographs showing only the eye region of faces expressing specific emotions. Participants choose the word that best describes the emotion in the main experimental condition of the RMET (hereafter, the RME task), while control conditions involve gender or age identification. fMRI research consistently highlight RME task-associated increased activation in ToM network areas, such as the bilateral temporo-parietal regions, including the pSTS, angular and supramarginal gyri (AG and SMG), as well as the IFG and middle frontal gyrus (MFG) (Baron-Cohen et al. 1999, Adams *et al.* 2010, Castelli *et al.* 2010, Gunther Moor *et al.* 2012, Overgaauw *et al.* 2015, Thye *et al.* 2018, Kamashita *et al.* 2024). Meta-analyses further corroborate these findings, also implicating the cingulate cortex/SMA and the left precentral gyrus (Schurz *et al.* 2014, Molenberghs *et al.* 2016). Importantly, the test’s wide applicability has been accompanied by ongoing discussion regarding the interpretation of its results.

For example, RMET was initially developed for autistic adults (Peñuelas-Calvo *et al.* 2019); however, later research has questioned whether autistic people lack ToM and therefore should perform worse on the RMET (Gernsbacher and Yergeau 2019, Silverman 2022). RMET has also been reported to be limited in differentiating between neuropsychiatric conditions, their stages and severity (Pavlova and Sokolov 2022). At the same time, it reliably differentiates between clinical and healthy populations across a variety of conditions (Johnson *et al.* 2022), including schizophrenia, depression, borderline personality disorder, neurodegenerative diseases, and eating disorders (Simon et al. 2019, Bora 2021, Preti *et al.* 2022, Stafford *et al.* 2023, Deng *et al.* 2024).

More broadly, the discussion has been raised regarding general use of the RMET. Studies often fail to provide test reliability and construct validity evidence (Higgins *et al.* 2024) and when reported, it does not always meet psychometric standards, including low internal consistency and uncertain factor structure (Olderbak *et al.* 2015, Higgins *et al.* 2023, 2025b, Van Staden and Callaghan 2022). These concerns have led to debate about whether the RMET should be abandoned (Higgins *et al.* 2025a), although this view has been contested (Murphy and Hall 2024).

Crucial for the interpretation of neuroimaging findings is a question of what the RMET actually measures (Pavlova and Sokolov 2022). The line of criticism highlights low convergent validity, with low or non-significant correlations between RMET scores and other measures of social cognition (Olderbak *et al.* 2015, Gernsbacher and Yergeau 2019), and limited discriminant validity, particularly between ToM, emotion recognition, and empathy (Oakley *et al.* 2016, Kittel *et al.* 2022, Pavlova and Sokolov 2022). RMET scores may also be influenced by demographic, socioeconomic and nonsocial factors, including social class (Dodell-Feder *et al.* 2020), IQ scores (Peñuelas-Calvo *et al.* 2019), working and episodic memory (Pavlova and Sokolov 2022), vocabulary and language skills (Gernsbacher and Yergeau 2019, Kittel *et al.* 2022, Silverman 2022). Accordingly, RME task-related neural activity in the left IFG may be interpreted as supporting language and semantic processing, rather than classical mentalizing (Pavlova and Sokolov 2022).

Nevertheless, the RMET remains widely used in both clinical and healthy populations, including studies of collective intelligence, stress, aging, and sex differences (Castelli *et al.* 2010, Hartshorne and Germine 2015, Woolley *et al.* 2015, Tollenaar and Overgaauw 2020, Greenberg *et al.* 2023). Its continued use is supported by multiple culturally adapted versions (Adams *et al.* 2010, Handley *et al.* 2019, Koo *et al.* 2021, Kim *et al.* 2024), which partially address cultural influences on performance (Dodell-Feder *et al.* 2020, Van Staden and Callaghan 2022).

Taken together, while the RMET has recognized limitations, its extensive validation across populations and widespread use in neuroimaging research make it a suitable and relevant paradigm for investigating neural correlates of mentalizing.

### BOLD signal decreases below baseline during the RME task

The modified and expanded Russian-language version of RMET comprising 144 stimuli developed by our group was used as a task design for the current study (Zheltyakova *et al.* 2025b). This version of RMET was also psychophysiologically tested through an fMRI study, revealing brain activity patterns similar to traditional neuroimaging findings associated with the RME task (Zheltyakova *et al.* 2025a). Our results replicate the literature findings, showing that the RME task (relative to a control task of age identification) is associated with increased BOLD signals in the bilateral IFG, pSTS, SMA, and the left precentral gyrus.

Additionally, a more detailed analysis revealed that brain regions exhibiting increased BOLD signal in the RME task compared to the control condition were not uniformly characterized by a simple increase in neural activity relative to baseline. Instead, we observed heterogeneous patterns, including (i) increased activation during the RME task, (ii) reduced deactivation compared to the control (in the left TPJ, including pSTS), and (iii) increased activation relative to deactivation during the control task (in the right TPJ). Additionally, brain areas such as the mPFC, precuneus, and AG, which typically feature in ToM-related activity, demonstrated significantly higher deactivation below baseline during the RME task compared to the age identification task, a pattern not previously reported in RMET-based neuroimaging studies.

Given that the relationship between the “below-baseline BOLD effect” of some ToM network regions and the socially relevant information processing remains unclear, the objective of the present study was to address this issue. This effect could signify task-related inhibition of the corresponding brain regions, reflecting a more complex redistribution of neural activity across the ToM network. Despite their deactivation, these regions may still contribute to affective state recognition, suggesting a form of functional involvement not captured by a straightforward activation approach.

### Task-modulated functional connectivity analysis

To complement and explain local BOLD signal changes, we propose performing task-modulated functional connectivity (TMFC) analysis of fMRI data gathered during the RMET. Considering the joint functioning of multiple distantly located areas across the whole brain, in addition to the functional activity of individual areas, has contributed to understanding the neural basis of social interactions (Maliske and Kanske 2022). Previous work has demonstrated that complex social tasks are associated with the integration of both affective and cognitive ToM network subcomponents, reflecting their interaction at the neural level and the combination of involved processes at the behavioral level. However, these conclusions have been based on indirect analyses of interactions, such as clustering analyses of activations (Schurz *et al.* 2021), analyses of co-activations (Maliske *et al.* 2023), or the joint activation of areas referring to different resting-state networks (Schurz *et al.* 2020). Few studies in the field have reported task-modulated effective (include the direction of influence) connectivity findings between areas from different socially relevant networks. In their review, Schurz *et al.* (2020) suggested two types of cross-network interactions: negative coupling and positive coupling between different networks, reflecting dynamic adaptation depending on the context and type of task.

Although such studies provide crucial information, connectivity studies targeting RME task performance remain scarce and primarily report effects of interventions, trauma, or clinical conditions (Nolte *et al.* 2013, van Schie *et al.* 2017), as well as analyze resting-state functional connectivity (Wang *et al.* 2016, Yin *et al.* 2018, Ilzarbe *et al.* 2019, 2021, Olivito *et al.* 2022). One study used independent component analysis, a network-level analysis that cannot demonstrate the role of individual nodes (Thye *et al.* 2018). To our knowledge, only two studies have reported functional interactions in healthy participants during RME task compared to a control task: one using psychophysiological interaction (PPI) analysis of fMRI data reported an interaction of the left IFG with the left inferior parietal lobule, lingual gyrus, and right SMA/ACC (Bos *et al.* 2016), and another using functional connectivity analysis of fNIRS data reported connections within the mPFC and between the mPFC and the dorsolateral PFC regions in the left hemisphere (Ieong and Yuan 2018). However, this evidence remains limited, and all regions of interest (ROI) in the named studies were situated in brain areas showing increased activation.

Another critical limitation of the majority of human brain imaging studies using RMET (including those reporting TMFC results) is the small number of unique stimuli (36 in the original study). According to our recent simulation study (Masharipov *et al.* 2024), at least 80 unique stimuli per condition are needed to ensure adequate statistical power in fMRI paradigms and to achieve >80% sensitivity in revealing changes in TMFC. The developed expanded Russian-language version of RMET comprising 144 stimuli overcomes this limitation (Zheltyakova *et al.* 2025b), providing sufficient data for examining TMFC with adequate specificity and sensitivity.

To address these issues, we analyzed TMFC utilizing generalized psychophysiological interaction analysis (gPPI) (Gitelman *et al.* 2003, McLaren *et al.* 2012, Masharipov *et al.* 2024) of fMRI data gathered during the RMET. This type of analysis estimates dynamic changes in functional connectivity between pre-selected (i.e., based on analyses of BOLD signal changes) areas of interest and all other voxels in the brain during one experimental condition compared to another. Thus, it provides an opportunity to understand how brain regions interact in a task-dependent manner (McLaren *et al.* 2012). Moreover, this approach is promising for evaluating the functional significance of below-baseline BOLD changes, as it has been shown that local activity and distant functional interactions do not always align (Kireev *et al.* 2015, Gerchen and Kirsch 2017, Satake *et al.* 2024). This was evidenced in micro-level studies of integrated neuronal population activity (Bechtereva 1974) and later confirmed at the macro-level of BOLD signal changes (Medvedev *et al.* 2019, 2023).

Specifically, regions that were deactivated during a social interaction task were nevertheless identified as related to task processing based on observed TMFC changes with other brain regions (Di and Biswal 2019, Medvedev *et al.* 2023). In original activation studies using the same experimental task with video clips of objects (squares, circles, triangles) either interacting in some way (social condition), or moving randomly (control condition), the social condition compared to control was associated with increased BOLD signal in ToM network structures, including the mPFC, TPJ, and superior and inferior temporal gyri (Castelli *et al.* 2000, Wheatley *et al.* 2007). Combined BOLD-signal and TMFC analysis has also revealed additional regions outside classically associated with social interactions showing decreased activity but increased functional connectivity, including the right cuneus, left precuneus, postcentral gyrus, and cerebellum (Di and Biswal 2019). Such areas with decreased BOLD signal that were functionally connected with other regions accounted for approximately 4.2% of analyzed nodes in the task network in this social cognition task (Medvedev *et al.* 2023). Thus, these two types of analyses can complement each other, reflecting distinct aspects of neural processing and revealing a broader task-related network, potentially including regions that initially show deactivation during RME task.

Taken together, the aim of the present study is to determine whether ToM network brain areas with BOLD signal changes below baseline during the RME task play a functional role in the neural processing of this social task. We hypothesize that, supplementing the analysis of local activity changes with gPPI analysis could clarify this issue, namely, TMFC changes would signify that these regions are in fact integrated into the task-relevant network, whereas the absence of TMFC changes would signify that they are actively disengaged from functional coupling with the involved brain network.

## Materials and methods

### Participants

In total, 65 volunteers, all right-handed native Russian speakers without any history of neurological or psychiatric disorders, current medication intake, and MRI contraindications, participated in fMRI recording while performing the modified version of RMET. We assessed the handedness of the participants using the Edinburgh Handedness Inventory (Oldfield 1971). All participants provided written informed consent prior to commencing the study. We performed all procedures in accordance with the Declaration of Helsinki, and they were approved by the Ethics Committee of the N.P. Bechtereva Institute of the Human Brain, St. Petersburg, Russia. After the study, all participants received a monetary reward (1500 rubles).

Five volunteers were subtracted from the analysis due to the low accuracy of the RME task (below 25%), leaving 60 participants (18 men, 42 women, age 23.4 ± 4 years).

### Stimuli and procedure

We used a modified and extended Russian-language adaptation of the RMET (Zheltyakova *et al.* 2025b). During the experimental condition (RME task) participants viewed photographs of the eye region presented in the center of the screen, along with four adjectives in each corner (e.g. “arrogant,” “depressed,” “confident,” “content”). Their task was to select the word that best described the expression (and, accordingly, the emotional state) of the person shown. The photographs were initially sourced from a stimulus bank collected at McGill University featuring two actors (one male, one female), then cropped, edited to depict only the eye region, and desaturated (Schmidtmann *et al.* 2020). Adjective options were fixed for each photograph (one correct answer and three fixed alternatives). As a control condition (AGE), photographs of the eye region were presented together with four age options (“twenty,” “thirty,” “forty,” “fifty,” “sixty”), and participants were instructed to determine the age of the person in the image. The stimulus set included only two actors, resulting in effectively two correct responses in the control condition: “twenty” for the female actor and “thirty” for the male actor. Although participants were not informed about the number of actors prior to scanning, this could become apparent during task performance. As a result, some participants adopted response strategies (e.g. consistently selecting the same option), which could lead to floor-level accuracy without necessarily reflecting inattentiveness.

The experiment included 144 original stimuli with four variants of presenting emotions: male or female face, and full-face or three-quarter view. The number of female faces was 74, compared to 70 male faces. Female images also included equal numbers of gazes of both viewing angles (37 each), whereas in male images, the direct angle predominated (39 vs. 31). Each stimulus was presented twice—once in the emotion recognition task and once in the age estimation task. The stimuli were not repeated under the same condition, resulting in a total of 288 trials.

**Figure 1:**
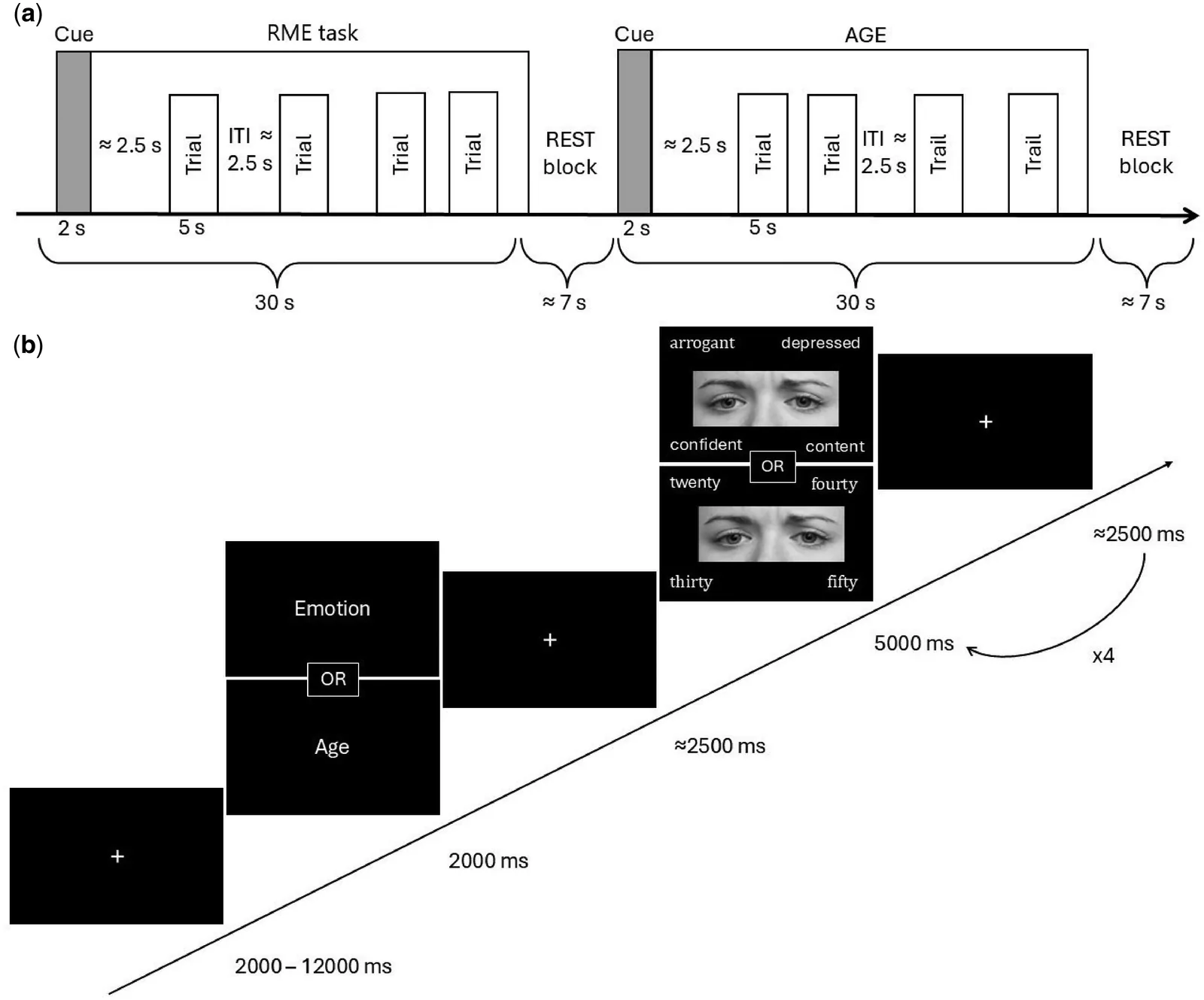
(a) Schematic representation of the experimental procedure; (b) illustration of experimental trials for the RME task (affective mental states recognition) social condition and the AGE (age identification) control condition.

The test consisted of three sessions, each lasting 17 minutes. Before each session, a fixation cross (a white cross on a black background) was shown for 5.5 s. Each session included alternating task blocks and rest blocks with fixation cross presentation (see Fig. 1). One session contained 24 task blocks and 24 rest blocks, with 12 blocks per condition (RME task and AGE).

Before the start of each task block, an instruction word (“emotion” or “age”) was displayed for 2 s. A fixation cross then appeared for a variable duration between 1.5 and 3.5 s (step = 0.5 s). Each task block consisted of four trials, each lasting 5 s. During each trial, participants viewed an eye region photograph with four response options (adjectives or ages) and selected an answer using a joystick button press. The intertrial interval varied between 1 and 4 s (step = 1 s), resulting in a total task block duration of 30 s. Rest blocks were presented between task blocks, with durations varying from 2 to 12 s (step = 2 s).

Stimulus presentation, response, and reaction time recording, as well as synchronization with fMRI image acquisition, were implemented using the NordicNeurolab head-mounted MR-compatible binocular goggle system and E-Prime 2.0 software (Psychology Software Tools Inc., Pittsburgh, PA, USA). The system’s diopters were adjusted for each participant to ensure a sharp image. Eye region stimuli size was 500 × 208/209 pixels and scaled to fit the display.

### fMRI image acquisition procedure and image processing

The experiment was conducted using a Philips Achieva 3.0 T MRI scanner. High-resolution structural data were obtained with a T1-weighted 3D fast field echo (T1W-3DFFE) sequence (repetition time, TR = 2.5 ms; echo time, TE = 3.1 ms; flip angle = 30°). The acquisition comprised 130 axial slices with a thickness of 0.94 mm and an in-plane resolution of 1 × 1 mm (field of view, FOV = 240 × 240 mm; matrix = 256 × 256). Functional images were collected using a single-shot echo-planar imaging (EPI) sequence sensitive to T2* contrast (TE = 35 ms; flip angle = 90°; FOV = 208 × 208 mm; matrix = 128 × 128). Each volume consisted of 32 axial slices, 3.5 mm thick (voxel size = 3 × 3 × 3.5 mm), covering the entire cerebral cortex and most of the cerebellum. Slices were aligned to match the orientation of the structural images, and functional volumes were acquired continuously with a TR of 2000 ms.

Preprocessing included motion correction, slice-timing adjustment, coregistration of functional and anatomical scans, segmentation of structural images into tissue classes, normalization to the Montreal Neurological Institute (MNI) standard space, and spatial smoothing using an 8 mm full-width-at-half-maximum Gaussian kernel. All image processing and statistical analyses were carried out in SPM12 (Statistical Parametric Mapping; Wellcome Department of Imaging Neuroscience, London, UK; http://www.fil.ion.ucl.ac.uk/spm) implemented in MATLAB (MathWorks, Natick, MA, USA). During scanning, participants’ head motion was minimized using an MRI-compatible Shantz collar for head and neck stabilization.

### Statistical analysis

To reveal brain structures with different types of effects (e.g. above and below the baseline) associated with the RME task the finite impulse response (FIR) analysis was used.

First, general linear models (GLMs) were created for each participant separately. They included regressors corresponding to experimental blocks: RME task and AGE trials. FIR analysis allows to estimate the time-course of event by dividing the course of each experimental condition into time bins and adding a separate regressor for each of these time points to the model. For the current analysis, each event block was modeled using a 40 s time window starting 2 s prior to block onset, with activity estimated every 2 s. This resulted in 20 regressors per condition (80 FIR regressors in total). To account for potential overlap between neighboring events, an additional canonical HRF-convolved cue regressor was included. This regressor modeled all cue events with onsets at cue presentation and a duration of 2 s. It was extracted from a separate GLM and subsequently added to the FIR model to partial out variance associated with the standard hemodynamic response. GLMs also included twenty-four regressors representing head movement parameters obtained during preprocessing (realignment; Friston *et al.* 1996). All first-level models were estimated using the robust weighted least squares (rWLS) toolbox implemented in SPM. The precision of the hyperprior used in the ReML (Restricted Maximum Likelihood) estimation within the rWLS framework was increased from 1/256 to 1/32. This modification improved numerical stability and produced a more reliable fit to local noise fluctuations.

Second, the beta values of the regression coefficients for the regressors in the GLMs were estimated at the individual level of analysis. Linear contrasts of the beta coefficients of each of the 80 FIR regressors and the baseline were calculated and used as a variable for the second-level analysis (representing BOLD signal difference between task performance and baseline, see Fig. 1a). For the second-level random-effect analysis, a flexible factorial design was used. The models included two factors: “task” (RME task or AGE) and “time bin” (20 levels). The interaction between factors was modeled to assess condition-dependent temporal dynamics of the response.

Finally, for a voxel-wise statistical inference at the group level and creation of plots of effect sizes for conditions of interest we specified t-contrasts aggregating across all time bins: (i) testing for overall activation above baseline; and (ii) testing for overall deactivation relative to baseline. Recomputed for 60 participants analog of previously obtained mask RME task > AGE was applied in both cases (Zheltyakova *et al.* 2025a) [for further information, see Supplementary Material 1, Table S1 (see online supplementary material for a color version of this table), and Figure S1 (see online supplementary material for a color version of this figure)]. The voxel-wise FWE corrected *P* < .05 threshold and the cluster size threshold = 10 were applied.

### ROI selection

We next pursued an assessment of TMFC between areas showing task-related BOLD signal changes. When selecting ROIs for this analysis, we considered that a single cluster of BOLD signal changes may encompass multiple anatomical and functional areas, each with distinct connectivity profiles.

Standard ROI selection approaches, which define a spherical ROI centered on the local maximum (or individual local maximum), have limitations related to unprecise anatomical identification. Specifically, local maxima can fall in different anatomical or functional areas across individuals, or on the borders between areas, rendering results incompatible, incomplete, and nonrepresentative.

To address these issues, our ROI selection procedure incorporated anatomical information using the Brainnetome Atlas (Fan *et al.* 2016). The Brainnetome Atlas provides a connectivity-based parcellation of the human brain into 210 cortical and 36 subcortical subregions and contains detailed information on both anatomical and functional connections. Moreover, resting-state fMRI analyses using the Brainnetome Atlas for brain parcellation have demonstrated an improved classification of functional states, such as eyes open and eyes closed during the resting state (Medvedeva *et al.* 2026), highlighting that connectivity-based parcellation provides more functionally meaningful ROIs compared to traditional anatomical or spherical approaches (Fan *et al.* 2016, Wu *et al.* 2025).

Accordingly, ROIs for the TMFC analysis were defined through a multi-step procedure: (i) brain regions showing significantly different BOLD signal during the RME task compared to the AGE condition (RME task > AGE) were defined; (ii) within these regions FIR analysis was used to separate clusters showing significant above- and below-baseline BOLD signal changes; and (iii) these clusters were further subdivided of into finer areas by masking them with the Brainnetome Atlas parcellation. Only those voxels that overlapped between the Brainnetome regions and clusters of observed significant task effects were retained for the subsequent seed-to-voxel TMFC analysis. Areas smaller than 20 voxels were excluded from the analysis.

### TMFC analysis

The generalized form of psychophysiological interaction analysis (gPPI; McLaren *et al.* 2012) with deconvolution (Gitelman *et al.* 2003) was used to assess TMFC, since according to our simulation study, it is the most sensitive method for block-design paradigms (Masharipov *et al.* 2024). gPPI analysis was conducted using the TMFC toolbox (https://github.com/IHB-IBR-department/TMFC_toolbox).

In the gPPI analysis, individual GLMs included the following regressors. First, psychological regressors representing task conditions were included in the gPPI model (RME task and AGE trials) using a FIR approach. This gPPI-FIR model provided better control for task-related co-activations with arbitrary HRF shapes (Cole *et al.* 2019, Masharipov *et al.* 2024). For each block, the time window was set to 30 s, starting 2 s before the onset of the block, and activity was estimated every 2 s.

Second, time series from each seed ROI were extracted as the first eigenvariate after removing the effects of no interest and applying whitening and high-pass filtering. Third, PPI regressors were calculated separately for each condition of interest. The computation included three steps. The BOLD signal time series from each ROI were deconvolved with the canonical HRF to estimate the underlying neuronal activity (Gitelman *et al.* 2003). Next, the deconvolved physiological signal was multiplied by the condition-specific psychological regressors (unconvolved event time courses), producing the PPI regressor at the neuronal level. This product was then reconvolved with the HRF to model the interaction at the BOLD level. The task design regressors were mean-centered prior to PPI term computation (Di *et al.* 2017, Masharipov *et al.* 2024). A whitening inversion procedure was applied to prevent double prewhitening of the seed regressor (He *et al.* 2025).

The gPPI analysis was performed in a seed-to-voxel manner, meaning TMFC was examined between each seed ROI and all other voxels across the brain. The beta values of the PPI regression coefficients were estimated at the individual level, and the RME task blocks were contrasted with the AGE blocks. The computed linear contrasts RME task >AGE and AGE >RME task entered the second-level group analysis. Group-level random-effects analyses were performed using a one-sample t-test. A voxel-wise uncorrected threshold of *P* < .001 and a cluster-wise FWE-corrected threshold of *P* < .05 were applied. Additional analyses assessing the potential influence of between-condition response time differences and individual RME task accuracy on the observed TMFC changes, yielded results consistent with the initial findings. Thus, only the main results are reported below. For further details on additional analyses and results, see Supplementary Material 5 and Table S7 (see online supplementary material for a color version of this table).

## Results

### Behavioral results

**Figure 2:**
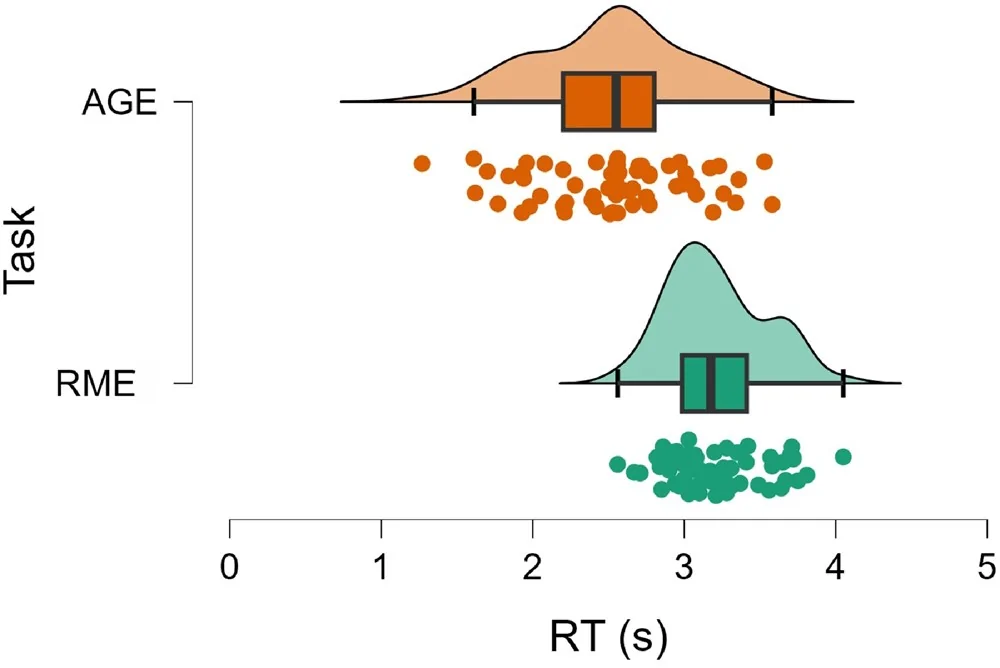
Raincloud plots of response time for the RME task and AGE conditions.

There was a statistically significant (*P* < .001) difference between response time for task conditions (RME task—3212 ± 322 ms, AGE condition—2526 ± 508 ms). The corresponding raincloud plots are presented in Fig. 2. The mean accuracy for the RME task was 67 ± 6.8%. Accuracy for the AGE control condition is not reported, as it is not straightforward to interpret due to task-specific response strategies (see Materials).

### Neuroimaging results

#### BOLD signal changes during mind reading from the eyes

Two aspects of BOLD signal are considered in the present study: (i) relative differences between conditions (RME task vs. AGE), and (ii) signal direction relative to baseline (above or below baseline). For the sake of clarity throughout the current manuscript the TMFC changes will be denoted as RME task-induced coupling and decoupling reflecting corresponding relative TMFC increases and decreases associated with the RME task in comparison with control one (AGE).

**Figure 3:**
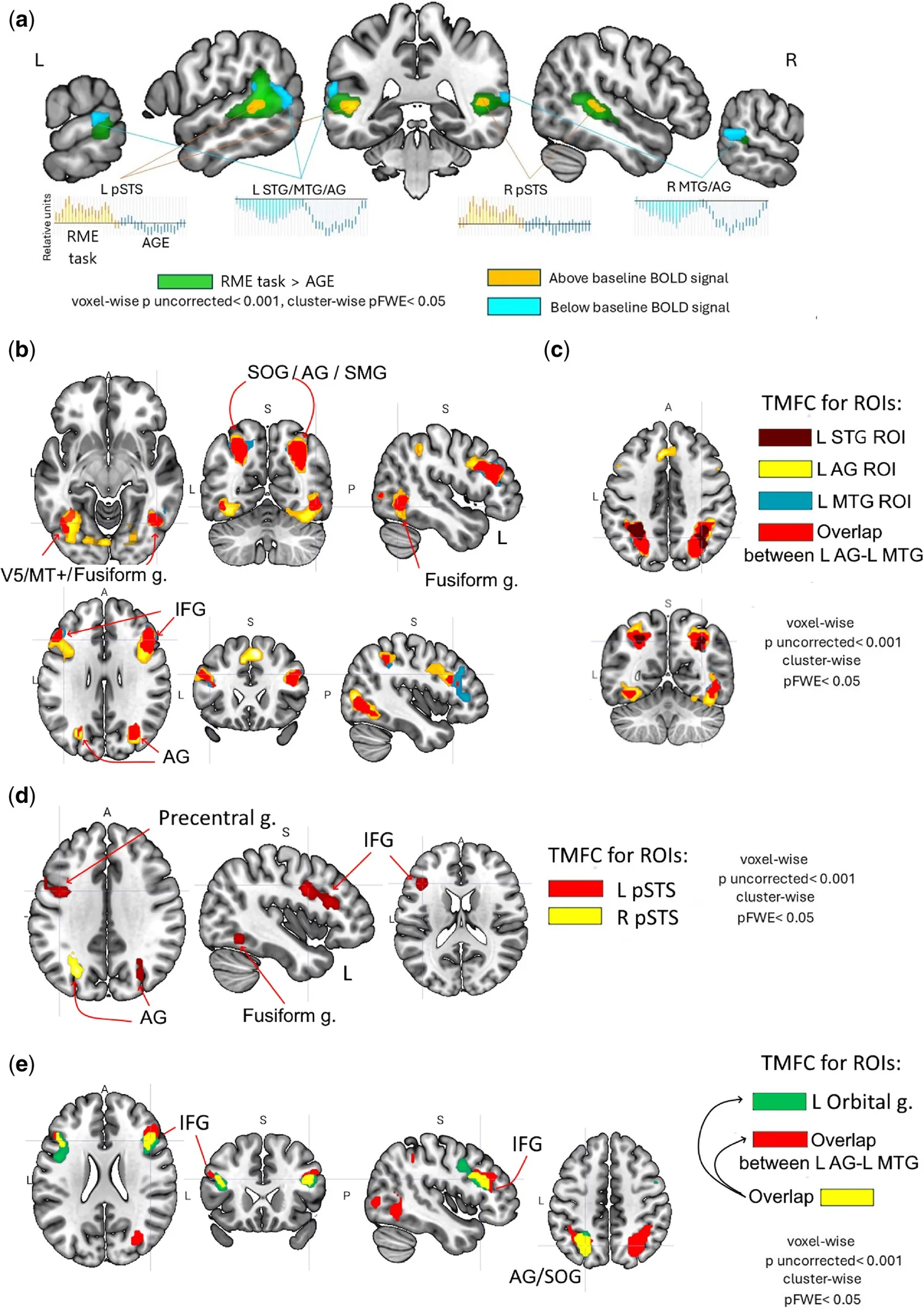
(a) Clusters identified by FIR analysis located within the TPJ-pSTS region defined by the standard analysis showing increased BOLD signal during recognition of affective mental states from the eyes (RME task) compared to age identification (AGE) (including subregions exhibiting increased activation above baseline and subregions, BOLD responses in which remain below baseline). The plots show effect sizes for the regressors in the FIR models (vs. baseline) with 90% confidence intervals; (b) Clusters showing RME task-induced increases of TMFC with the left AG and MTG subregions from the TPJ-pSTS region that showed greater BOLD response during the RME task (vs. AGE), despite this response remaining *below* baseline; (c) Clusters showing RME task-induced increases of TMFC with the left AG, MTG, and STG subregions from the TPJ-pSTS region that showed greater BOLD response during the RME task (vs. AGE), despite this response remaining *below* baseline; (d) Clusters showing RME task-induced increases of TMFC with the right and left pSTS subregions from the TPJ-pSTS region that showed greater above-baseline activation during the RME task (vs. AGE); (e) Clusters showing RME task-induced increases of TMFC with the left orbital gyrus subregion outside the TPJ-pSTS region that showed greater above-baseline activation during the RME task (vs. AGE). Alt text: Figure 3 (a) Clusters from FIR analysis within TPJ-pSTS showing increased BOLD signal during RME > AGE; (b) Clusters showing RME-induced increases in TMFC with left AG/MTG; (c) left AG/MTG/STG; (d) bilateral pSTS; (e) and left orbital gyrus.

First, we examined regions showing greater BOLD responses during the RME task compared to the AGE condition (RME task > AGE) [Figure S1 (see online supplementary material for a color version of this figure) and Table S1 (see online supplementary material for a color version of this table)]. Using FIR analysis within the corresponding clusters, we identified that these regions exhibited both above- and below-baseline BOLD signal changes. Specifically, within the left and right TPJ-pSTS region, some areas showed above-baseline activation during the RME task, particularly the bilateral pSTS. In contrast, other subregions, including dorsolateral parts of the bilateral middle temporal gyrus (MTG, BA 37), rostroventral parts of the bilateral AG (BA 39), and the caudal portion of the left superior temporal gyrus (STG, BA 22), showed greater responses during the RME task than AGE, yet these responses remained below baseline (see Fig. 3a and Table 1). Along with that, FIR analysis revealed a broader list of brain regions that showed stronger responses during the RME task compared to AGE [Figure S2 (see online supplementary material for a color version of this figure) and Table S2 (see online supplementary material for a color version of this table)].

**Table 1:** Regions identified by FIR analysis showing different types of BOLD signal changes relative to baseline within TPJ-pSTS cluster defined in the standard analysis of BOLD signal changes during the RME task compared to the AGE control condition (voxel-wise FWE-corrected *P* < .05, cluster size threshold = 10).

Region	BNT subregion	Cluster	Peak					
		*k*	*T*	*Z*	*P*(FWE-corr)	*x*	*y*	*z*
**RME task > AGE** **Above-baseline BOLD signal during the RME task**								
Left STG	Posterior STS	119	10.59	>8	<.001	−48	−43	5
			5.51			−39	−61	2
Right STG	Posterior STS	21	6.36	6.35	<.001	51	−37	5
**RMET task > AGE** **Below-baseline BOLD signal during the RME task**								
Left STG/MTG/AG	Dorsolateral area 37	92	10.65	>8	<.001	−60	−61	14
	Caudal area 22		9.91			−54	−67	11
	Rostroventral area 39 (PGa)		9.55			−63	−37	20
Right MTG/AG	Dorsolateral area 37	52	10.13	>8	<.001	66	−46	17
	Rostroventral area 39 (PGa)		9.85			63	−55	14

Each region is aligned with the corresponding subregions of the Brainnetome Atlas (Fan *et al.* 2016).

Further, based on the results of the FIR analysis, we created a set of ROIs for the TMFC analysis. These ROIs were defined using the Brainnettome atlas and included three groups that demonstrated greater BOLD responses during the RME task compared to the AGE condition (Figure S3, see online supplementary material for a color version of this figure): (i) located within the left and right TPJ-pSTS regions with below-baseline BOLD signal; (ii) located within the left and right TPJ-pSTS regions with above-baseline activation; (iii)located outside the left and right TPJ-pSTS regions.

#### RME task-induced TMFC changes

##### RME task-induced coupling of brain regions showing greater BOLD responses during the RME task, with responses remaining below baseline (ROIs within the TPJ-pSTS region)

ROIs located within the left and right TPJ-pSTS region that showed greater responses during the RME task than AGE, despite BOLD signal remaining below baseline, demonstrated only increased TMFC during the RME task as compared to the AGE condition (see Table 2).

**Table 2:** RME task-induced increases in TMFC for ROIs within the TPJ-pSTS region showing greater BOLD responses during the RME task, with responses remaining below baseline (voxel-wise uncorrected *P* < .001, cluster-wise FWE-corrected *P* < .05).

BNT region	BNT subregion	Cluster		Peak					
		*P*(FWE-corr)	*k*	*T*	*Z*	*P*(unc)	*x*	*y*	*z*
**TMFC increase for the ROI in the left MTG (dorsolateral area 37)**									
Right		<.001	1156	6.7	5.7	<.001	30	−61	38
Occipital g./	Middle occipital g.;								
	Area V5/MT+;								
	Occipital polar cortex;								
	Inferior occipital g./								
SOG/	Lateral SOG/								
AG/	Rostrodorsal area 39 (Hip3);								
	Caudal area 39 (PGp)/								
Fusiform g./	Medioventral area 37;								
	Lateroventral area 37								
	Inferior occipital g./								
SMG/	Rostrodorsal area 40 (PFt)/								
SPL	Intraparietal area 7(hIP3)								
	Caudal area 7;								
Left		<.001	999	5.1	4.9	<.001	−39	−76	2
Occipital g./	Middle occipital g.;								
	Area V5/MT+;								
	Occipital polar cortex;								
	Inferior occipital g./								
SOG/	Lateral SOG/								
Fusiform g./	Medioventral area 37;								
	Lateroventral area 37/								
AG/	Rostrodorsal area 39 (Hip3);								
	Caudal area 39 (PGp)/								
	(PFt)/								
SMG/	Rostrodorsal area 40								
SPL/	Intraparietal area 7 (hIP3);								
Cuneus/	Lateral area 5/								
	Caudal lingual g.								
Right		<.001	266	4.4	4.1	<.001	39	20	26
Precentr g./	Caudal ventrolateral area 6/								
IFG/	Dorsal area 44;								
	Inferior frontal sulcus;								
MFG	Ventral area 9/46/								
	Ventrolateral area 8;								
	Inferior frontal junction								
Left		<.001	235	4.3	4	<.001	−33	11	32
Precentr. g./	Caudal ventrolateral area 6/								
IFG	Dorsal area 44								
MFG	Inferior frontal junction								
Right/Left	Medial area 8	.002	177	5.8	5.2	<.001	6	20	50
SFG	Medial area 9								
**TMFC increase for the ROI in the left AG (rostroventral area 39 (PGa))**									
Right		.048	79	4.1	3.9	<.001	45	−58	−7
Occipital g./	Area V5/MT+/								
Fusiform g./	Lateroventral area 37								
Left		.019	103	4.1	3.8	<.001	−42	−73	−1
Occipital g./	Area V5/MT+/								
Fusiform g./	Lateroventral area 37								
Right		<.001	588	6.1	5.4	<.001	30	−58	35
Occipital g./	Middle occipital g.;								
	V5/MT+								
SOG/	Lateral SOG/								
AG/	Rostrodorsal area 39								
SMG/	(Hip3)/								
SPL	Rostrodorsal area 40 (PFt)/								
	Caudal BA 7;								
	Intraparietal area 7 (hIP3)								
Left		.008	127	4.7	4.4	<.001	−48	32	32
IFG/	Inferior frontal sulcus/								
MFG	Ventral area 9/46								
Right		.001	180	4.7	4.3	<.001	48	32	26
IFG/	Dorsal area 44;								
MFG	Inferior frontal sulcus/								
	Ventral area 9/46;								
	Ventrolateral area 8								
Left		<.001	299	5.2	4.7	<.001	−30	−64	44
SOG/	Lateral SOG/								
AG/	Rostrodorsal area 39 (Hip3);								
SPL	Caudal area 39 (PGp)/Intraparietal area 7 (hIP3);								
	Postcentral area 7;								
	Lateral area 5								
**TMFC increase for the ROI in the left STG (caudal area 22)**									
Right		.018	109	4.3	3.9	<.001	36	−52	44
AG/SOG	Rostrodorsal area 39 (Hip3)/								
	Lateral SOG								
Left		.042	85	4.1	3.8	<.001	−33	−49	44
AG/SPL	Rostrodorsal area 39 (Hip3)/								
	Lateral area 5;								
	Intraparietal area 7 (hIP3)								
**TMFC increase for the ROI in the right MTG (dorsolateral area 37)**									
Right		<.001	237	4.9	4.4	<.001	33	−58	41
AG/	Rostrodorsal area 39 (Hip3)/								
SMG/	Rostrodorsal area 40 (PFt)/								
SOG	Lateral SOG								
**TMFC increase for the ROI in the right AG (rostroventral area 39 (PGa))**									
Right		<.001	246	4.5	4.2	<.001	30	−58	38
AG/SMG/	Rostrodorsal area 39 (Hip3) Rostrodorsal area 40 (PFt)/								
SOG	Lateral SOG								

Each region is aligned with the corresponding subregions of the Brainnetome Atlas (Fan *et al.* 2016).

Overlapping patterns of RME task-induced coupling were observed for ROIs located in the MTG and AG subregions of the left TPJ-pSTS region (see Fig. 3b). Their TMFC was increased with areas located bilaterally in the fusiform gyri (lateroventral area 37), middle and inferior occipital gyri, V5/MT+ areas, lateral superior occipital gyri (SOG), AG (rostrodorsal area 39 (Hip3) and caudal area 39 (PGp)) and SMG (rostrodorsal area 40 (PFt)), SPL (bilateral intraparietal area 7 (hIP3), right caudal area 7, and left lateral area 5), MFG, including subregions in the ventrolateral area 8 and ventral area 9/46, and IFG, including subregions in dorsal area 44 and inferior frontal sulcus. For the adjacent ROI in the caudal area 22 of the STG (located within the left TPJ-pSTS region), RME task-induced coupling was revealed with a more restricted set of brain regions in the right lateral SOG, left superior parietal lobule (SPL), including lateral area 5 and intraparietal area 7 (hIP3), and rostrodorsal area 39 (Hip3) of the left and right AG (see Fig. 3c). Likewise, two ROIs from the MTG and AG subregions of the right TPJ-pSTS demonstrated a convergent pattern of RME task-induced TMFC increase with one cluster located in the right lateral SOG, rostrodorsal area 39 (Hip3) of AG, and rostrodorsal area 40 (PFt) of SMG.

##### RME task-induced coupling of brain regions showing above-baseline activation during the RME task (ROIs within the TPJ-pSTS region)

For the left pSTS ROI, increased TMFC during the RME task (vs. AGE) was observed with brain areas including the left fusiform gyrus (including lateroventral and medioventral area 37), right lateral SOG, left caudal ventrolateral BA6 of precentral gyrus, and subregions of the left IFG in the dorsal area 44 and the inferior frontal sulcus (see Table 3 and Fig. 3d). This pattern of TMFC changes overlapped with the RME task-induced coupling revealed for the previously described ROIs in the left AG and MTG subregions of the left TPJ-pSTS region.

**Table 3:** RME task-induced increases in TMFC for ROIs within the TPJ-pSTS region showing above-baseline activation during the RME task (voxel-wise uncorrected *P* < .001, cluster-wise FWE-corrected *P* < .05).

BNT region	BNT subregion	Cluster		Peak					
		*P*(FWE-corr)	*k*	*T*	*Z*	*P*(unc)	*x*	*y*	*z*
**TMFC increase for the ROI in the left pSTS**									
Left	Lateroventral area 37;	.01	120	5.3	4.8	.002	−36	−55	−13
Fusiform g.	Medioventral area 37								
Left	Caudal ventrolateral BA6/	.004	147	4.8	4.4	<.001	−42	−1	32
Precentr. g./IFG	Dorsal area 44; Inferior frontal sulcus								
Right SOG	Lateral SOG	.009	126	4.8	4.4	.001	33	−70	26
TMFC increase for the ROI in the right pSTS									
Left		.045	82	4.3	4.03	<.001	−30	−58	38
SOG/AG	Lateral SOG/ Caudal area 39 (PGp)								

Each region is aligned with the corresponding subregions of the Brainnetome Atlas (Fan *et al.* 2016).

Contrary to that, the right pSTS ROI demonstrated increased TMFC during the RME task (vs. AGE) only with the caudal area 39 (PGp) of the left AG, a pattern that was also observed for the AG and MTG subregions of the left TPJ-pSTS region (see Table 3 and Fig. 3d).

RME task-induced coupling of brain regions showing above-baseline activation during the rme task (ROIs Outside the TPJ-pSTS region).

Other ROIs that demonstrated significant TMFC changes were located outside the TPJ-pSTS region in the left temporal cortex, left orbitofrontal gyrus, right anterior insula, and bilateral IFG (Table S2, see online supplementary material for a color version of this table).

For the ROI in the lateral area 38 of the left STG, increased TMFC during the RME task (vs. AGE) was observed with the left and right AG and SMG, including rostrodorsal area 39 (Hip3) and rostrodorsal area 40 (PFt) respectively. These clusters of TMFC increases overlapped with those observed for the AG and MTG ROIs from the left TPJ-pSTS region (Table S3, see online supplementary material for a color version of this table). There was one nonoverlapping cluster of increased TMFC in the right caudal ventrolateral area 6 and inferior frontal junction from precentral gyrus and MFG.

For the ROI from the left orbital gyrus (lateral area 12/47), increased TMFC during the RME task (vs. AGE) was observed across multiple left hemisphere clusters: the precentral gyrus (caudal ventrolateral area 6), IFG (dorsal area 44), and SOG/IPL cluster, including the lateral SOG, Hip3 and PGp subregions of the AG [Table S3 (see online supplementary material for a color version of this table) and Fig. 3e]. In the right hemisphere only one cluster of increased TMFC was observed in the right prefrontal cortex in the caudal ventrolateral area 6 of precentral gyrus, dorsal area 44 of IFG, and inferior frontal junction of MFG.

The rest of the ROIs, located in the left IFG (the caudal area 45 and ventral area 44) demonstrated convergent TMFC increase during the RME task (vs. AGE) located in the left caudal ventrolateral area 6 of precentral gyrus and left area 44 (Table S3, see online supplementary material for a color version of this table). Finally, the ROI from the right caudal area 45 of the IFG increased its TMFC with the subregion of the left IFG in the caudal area 45.

##### RME task-induced decoupling of brain regions showing greater BOLD responses during the RME task

A substantial number of ROIs demonstrated reduced TMFC during the RME task as compared to the AGE condition with the brain regions located in the left IPL, bilateral precuneus, bilateral posterior and subgenual anterior cingulate gyrus, bilateral mPFC (including SFG and MFG) (Table S4, see online supplementary material for a color version of this table). Brain regions that demonstrated greater BOLD responses during the RME task (vs. AGE) within the rostral and caudal subregions of the area 45 of the left IFG and the left lateral area 12/47 of the left orbital gyrus were decoupled from the AG (PGa) and SMG (Pfm) subregions of the left TPJ, bilateral caudal and dorsal subregions of the area 23 in the posterior cingulate gyrus, area 31 (Lc1) subregion of the precuneus, and area 10 of the SFG. Other subregions of the left IFG located in the ventral, dorsal, and orbital area 44 showed similar patterns of reduced connectivity with the same subregions of the posterior cingulate/precuneus area and the SFG. Additionally, dorsal and ventral area 44 from the left IFG showed reduced connectivity with the AG (PGa) and SMG (Pfm) subregions of the left TPJ. Moreover, subcortical ROIs located in the left globus pallidus and the left medial prefrontal thalamus also showed convergent reductions in connectivity with the SFG. However, for the left globus pallidus, this reduction in connectivity was more widespread and extended to the anterior cingulate gyrus subregions in the left pregenual area 32 and the right caudodorsal area 24. Also, the ROI from the left dorsal agranular insula, together with the left globus pallidus, left dorsal area 44, and caudal area of the left IFG, showed reduced connectivity with posterior cingulate/precuneus subregions and subgenual area 32 of the cingulate gyrus. Other subregions, such as dorsal area 9/46 and area 46 located mainly within the left MFG, reduced connectivity with ROIs from the left globus pallidus, left caudal area 45, and opercular area 44 of the IFG.

## Discussion

The primary aim of the current study was to assess the functional role of the “below-baseline BOLD effect” in ToM network regions during socially relevant information processing. It was addressed by estimating the functional coupling of TPJ subregions that demonstrated greater BOLD responses during the RME task (vs. control age identification condition), with these responses remaining below baseline. As a result, it was for the first time revealed that, despite the below-baseline decrease of the BOLD signal in both RME task and control conditions, TPJ-pSTS subregions located in the left MTG, STG, and AG showed increased functional coupling during the RME task that converged in brain structures including the IFG, MFG, occipital cortex (with the occipital face area in SOG), V5/MT+, fusiform gyrus (with the fusiform face area), and parts of the AG, SMG, and SPL. A practically similar pattern of whole-brain TMFC changes was observed for a group of ROIs with above-baseline activations during the RME task. Together, ROIs showing both above-baseline activation and greater responses during the RME task (vs. AGE), with these responses remaining below baseline, and their overlapping patterns of TMFC formed an RME task-associated brain network. Supplementary analyses addressing the possible impact of response time difference between compared conditions and individual RME task accuracy reproduced these effects. Functional specialization of the revealed brain areas indicates that the RME task performance engages both affective mentalizing and visual face-processing mechanisms. Based on this finding, we can conclude that brain structures with RME task-related activity are involved in RME task processing irrespective of the direction of BOLD signal changes relative to baseline.

### RME task-induced coupling between RME task-associated brain network areas

The observed pattern of TMFC coupling aligns closely with the current knowledge on the neural correlates of the RME task. Meta-analyses have consistently implicated the bilateral pSTS and adjacent TPJ regions, IFG, MFG, left precentral gyrus, and SMA in the RME task processing and preferentially linked them to the affective subcomponent of the mentalizing neural network (Schurz *et al.* 2014, Molenberghs *et al.* 2016).

In addition, our results revealed the involvement of the bilateral occipital cortex, fusiform gyrus, and V5/MT+, i.e. brain regions known to be parts of the face-processing network specialized for the visual analysis of facial features, including emotional facial expressions (Haxby *et al.* 2000, O’Toole *et al.* 2002, Gobbini and Haxby 2007, Bernstein and Yovel 2015). The middle and superior occipital gyri correspond to the occipital face area, implicated in early facial perception, whereas the fusiform gyri correspond to the fusiform face area, a ventral-stream region processing structural information about facial form and features, while the motion-selective area MT is responsible for dynamic information processing, extending to facial movement analysis.

Previous research has shown that the pSTS contributes not only to affective mentalizing but also to dynamic aspects of face processing, such as gaze direction, facial expression, and lip movement (Haxby *et al.* 2000, Gobbini and Haxby 2007, Yang *et al.* 2015). Within this network, the pSTS acts as a hub for integrating motion-related facial cues from the MT and relaying them to anterior regions, including the aSTS and IFG, both of which have been identified as motion-sensitive face areas (Bernstein and Yovel 2015, Wang *et al.* 2016).

Importantly, some areas included in the RME task-associated brain network correspond to the principal hubs of the mirror neuron system (MNS), activated during observed and performed motor actions, but also implicated in higher-order social cognition, such as understanding emotions in humans and macaques (Rizzolatti *et al.* 2014, Jeon and Lee 2018). In particular, observing the emotions of others (including emotional facial expressions) is believed to activate the same emotion in the observer via a cortical mirror mechanism that transforms sensory information into one’s own somatomotor and visceromotor representations (Gallese *et al.* 2004). Namely, the ventral part of the prefrontal gyrus (caudal ventrolateral area 6) is a human homolog for area F5 of the ventral premotor cortex in macaques, where mirror neurons were first discovered (di Pellegrino *et al.* 1992, Gallese *et al.* 1996). Also, the specific observed part of the SMG (rostrodorsal area 40 (PFt) of the IPL) corresponds to the principal parietal hub of MNS in macaque brains (Fogassi and Luppino 2005, Fogassi *et al.* 2005). Furthermore, the STS is considered to be a structure of the MNS (Molenberghs *et al.* 2010). Summing up, this pattern supports the interpretation that RME task performance engages not only the mentalizing network but also neural systems underlying facial emotion decoding.

This aligns with earlier critiques suggesting that the RME task assesses emotion recognition abilities rather than exclusively ToM. Behavioral studies have shown that RME task performance correlates more strongly with individual differences in alexithymia (commonly associated with emotion recognition deficits) than with autism-spectrum traits (Oakley *et al.* 2016). Performance on the RME task also shows the strongest meta-analytic association with emotion perception, with only 15% of its variance shared with other ToM measures (Kittel *et al.* 2022). However, the RME task is also limited as a purely emotion perception measure.

An alternative, reconciling, view suggests that, rather than strictly contrasting ToM and emotion recognition, these processes may be closely related, partially overlapping, or even reflect a shared process relying on affective perceptual mechanisms captured by the RME task (Kittel *et al.* 2022). Within the ToM framework, mental state inference can be viewed as a two-stage process: first, mental state decoding, involving the identification of others’ thoughts or feelings based on external cues, followed by mental state reasoning, involving inference about why these mental states occur (Harkness *et al.* 2005). The first stage is closely related to emotion recognition, as both rely on external multimodal cues (Harkness *et al.* 2005, Young and Bruce 2011, Connolly *et al.* 2020), and there is no agreement on the hierarchy between them, i.e. which is a component of the other.

Thus, the psychometric design of the RME task, together with obtained evidence at the neural level, suggests that the test relies on a combination of emotion recognition and affective ToM.

Taken together, our findings indicate that RME task performance depends on the joint engagement of affective mentalizing and visual face- and emotion-recognition networks. The observed correspondence of increases in both local activity and inter-regional TMFC across these regions suggests that the RME task requires integration of internal affective representations with external sensory information.

### RME task-induced decoupling

Apart from coupling within the RME task-associated brain network, its areas demonstrated reduced functional connectivity with a number of areas, including the AG [rostroventral area 39 (PGa)] and SMG [caudal area 40 (PFm)] subregions of the left TPJ (outside the TPJ-pSTS region), precuneus, as well as dorsal and ventral mPFC during the RME task (vs. AGE). These regions are also core components of the ToM network (Mar 2011, Schurz et al. 2014, Molenberghs et al. 2016, Arioli et al. 2021, Diveica et al. 2021, Schurz et al. 2021, Balgova et al. 2024), especially its cognitive subcomponent. They also partially overlap anatomically with the default mode network (DMN) (Schilbach *et al.* 2008, Yeo *et al.* 2011, Mars *et al.* 2012, Schilbach *et al.* 2012, Bzdok *et al.* 2013, Spunt *et al.* 2015, Igelström and Graziano 2017). However, detailed analyses–including single-subject contrasts–indicate that ToM- and DMN-related subregions can be distinguished within these broader structures (Buckner *et al.* 2008, Spreng and Grady 2010, Andrews-Hanna *et al.* 2014, DuPre *et al.* 2016, Allen *et al.* 2017, Buckner and DiNicola 2019, DiNicola *et al.* 2020). For example, in the IPL, DMN-related tasks recruit more posterior regions, whereas ToM tasks engage anterior regions extending toward the TPJ. At the connectome level, these networks differ in connectivity, lateralization, and homogeneity: the DMN is left-lateralized and more uniform, while the ToM network is right-lateralized and functionally heterogeneous (Wang *et al.* 2021). Given the RME task reliance on inferring others’ emotions and the overlap between the meta-analytically outlined ToM network and the RME task-induced decoupling pattern, the observed regions can therefore be functionally attributed to the ToM rather than the DMN network (Schurz *et al.* 2021).

The obtained results are in line with the growing emphasis that effective social interaction requires both segregation (negative coupling) and integration (positive coupling) between brain networks in social cognition (Watanabe and Rees 2017, Schurz *et al.* 2020, Maliske and Kanske 2022, Czekóová *et al.* 2025). Importantly, earlier studies have made conclusions on ToM network functioning during RME task using an activation-based and meta-analytical approach (Schurz *et al.* 2021). Thus, to our knowledge, we provide the first TMFC-based results, demonstrating the dynamic interaction between ToM network subcomponents during the RME task compared to the control age identification condition: structures preferentially referred to the affective subcomponent form the RME task-associated neural network, while structures of the cognitive subcomponent decouple from the task-relevant brain network.

## Conclusion

The key finding of the current study is for the first time demonstrating that, despite their BOLD signal decrease below baseline, the TMFC profile of TPJ-pSTS subregions closely resembles RME task-induced functional connectomics of ToM network brain areas in the pSTS and IFG that exhibit standard above-baseline activations during the RME task. Although the neural mechanisms and physiological meaning underlying the below-baseline BOLD decreases remain unclear, the present results indicate that such areas are fully engaged in the network of task-relevant brain regions and are functionally integrated with them. In addition, the current study extends classical findings of activation-based and meta-analytical research by using TMFC analysis to demonstrate the dynamic interaction within the ToM network during the RME task in healthy participants, including the engagement of the affective ToM network subcomponent as well as its segregation from the cognitive ToM network subcomponent.

## Supplementary Material

nsag042_Supplementary_Data

## Data Availability

All data are available upon request.
