# Supplementary material for: Human brain network for reading the mind from the eyes: beyond activated and deactivated regions
Source: Soc Cogn Affect Neurosci. 2026 Jun 3;21(1):nsag042. doi: 10.1093/scan/nsag042 (PMC13285000; doi:10.1093/scan/nsag042)
Supplement: nsag042_Supplementary_Data [file nsag042_supplementary_data.zip › Supplementary_Materials.docx]

**Supplementary Materials**

1. **Significant BOLD Signal Changes, Associated with the RME Task**

First, we determined regions showing greater BOLD responses during the RME task compared to the AGE condition (RME task > AGE) to further apply them as a mask in the FIR-analysis. This is a recomputed analog (for 60 participants) of previously obtained results of RME task > AGE comparison (Zheltyakova et al., 2025a).

The analysis was performed using a standard approach. Individual level GLMs included two regressors representing two task conditions (blocks): (1) the RME task (social interaction condition) and (2) the AGE (control condition). Twenty-four motion parameters were included as nuisance regressors (Friston et al., 1996). Task regressors were created by convolving the canonical hemodynamic response function (HRF) with temporal characteristics of blocks (onset times at the beginning of the block). At the first level, RME task > AGE and Condition > Rest betas contrasts were created. At the second (group) level, a one-sample *t*-test was performed on the RME task > AGE betas contrasts to identify brain regions associated with the ToM-related social condition. Table S1 presents the results of this analysis obtained using classical frequentist statistics inference and a gray matter mask derived from individual T1 segmentation maps. Figure S1 further illustrates the results of the RME task > AGE comparison with two statistical thresholds.

Additionally, to assess the directionality of significant BOLD signal changes and create plots of effect sizes for conditions of interest, full-factorial model for betas revealed in RME task > Rest and AGE > Rest comparisons was estimated (see plots in Figure S1).

To further assess the potential influence of task difficulty and individual differences on the observed BOLD signal effects, second-level one-sample t-test models were re-estimated with response time (difference between conditions) and RME task accuracy (percent correct) included as covariates of no interest (see Supplementary Materials 5, Table S5 and Figure S4). The results were highly consistent with the original findings, further supporting their robustness.

**Table S1.** Clusters showing relative BOLD signal increases associated with recognizing affective mental states of others from the eyes (voxel-wise uncorrected p < 0.001, cluster-wise FWE-corrected p < 0.05, cluster size threshold = 10).

| **Anatomical localization** | **Cluster** | | **Peak** | | | | | |
| --- | --- | --- | --- | --- | --- | --- | --- | --- |
|  | **p(FWE-corr)** | **k** | **T** | **Z** | **p(unc)** | **x** | **y** | **z** |
| **RME task >AGE** | | | | | | | | |
| L IFG, Insula, Precentral g., Postcentral g., STG, MTG, Temporal pole, Superior/middle/inferior occipital g., Fusiform g., Lingual g., Calcarine g., Cerebellum, Putamen, Caudate n., Thalamus, Hippocampus  R STG, MTG, Temporal pole, Cuneus, Superior occipital g., Fusiform g., Lingual g., Calcarine g., Cerebellum, Caudate n., Thalamus, Hippocampus | <0.001 | 7164 | 7.52 | 6.27 | <0.001 | -54 | 20 | 17 |
|  |  |  | 7.34 | 6.16 |  | -57 | -46 | 11 |
|  |  |  | 7.31 | 6.14 |  | -39 | -7 | 41 |
| R IFG | <0.001 | 589 | 6.11 | 5.35 | <0.001 | 48 | 26 | -4 |
|  |  |  | 5.82 | 5.16 |  | 36 | 26 | -1 |
|  |  |  | 5.53 | 4.94 |  | 57 | 26 | 8 |
| L SMA | <0.001 | 578 | 5.83 | 5.16 | <0.001 | -6 | 17 | 44 |
|  |  |  | 5.47 | 4.90 |  | 0 | 8 | 59 |
|  |  |  | 5.12 | 4.64 |  | 9 | 2 | 50 |
| R Precentral g./MFG | <0.001 | 271 | 5.32 | 4.79 | <0.001 | 45 | -1 | 47 |
|  |  |  | 5.07 | 4.60 |  | 42 | -10 | 53 |
|  |  |  | 4.21 | 3.92 |  | 33 | -10 | 62 |


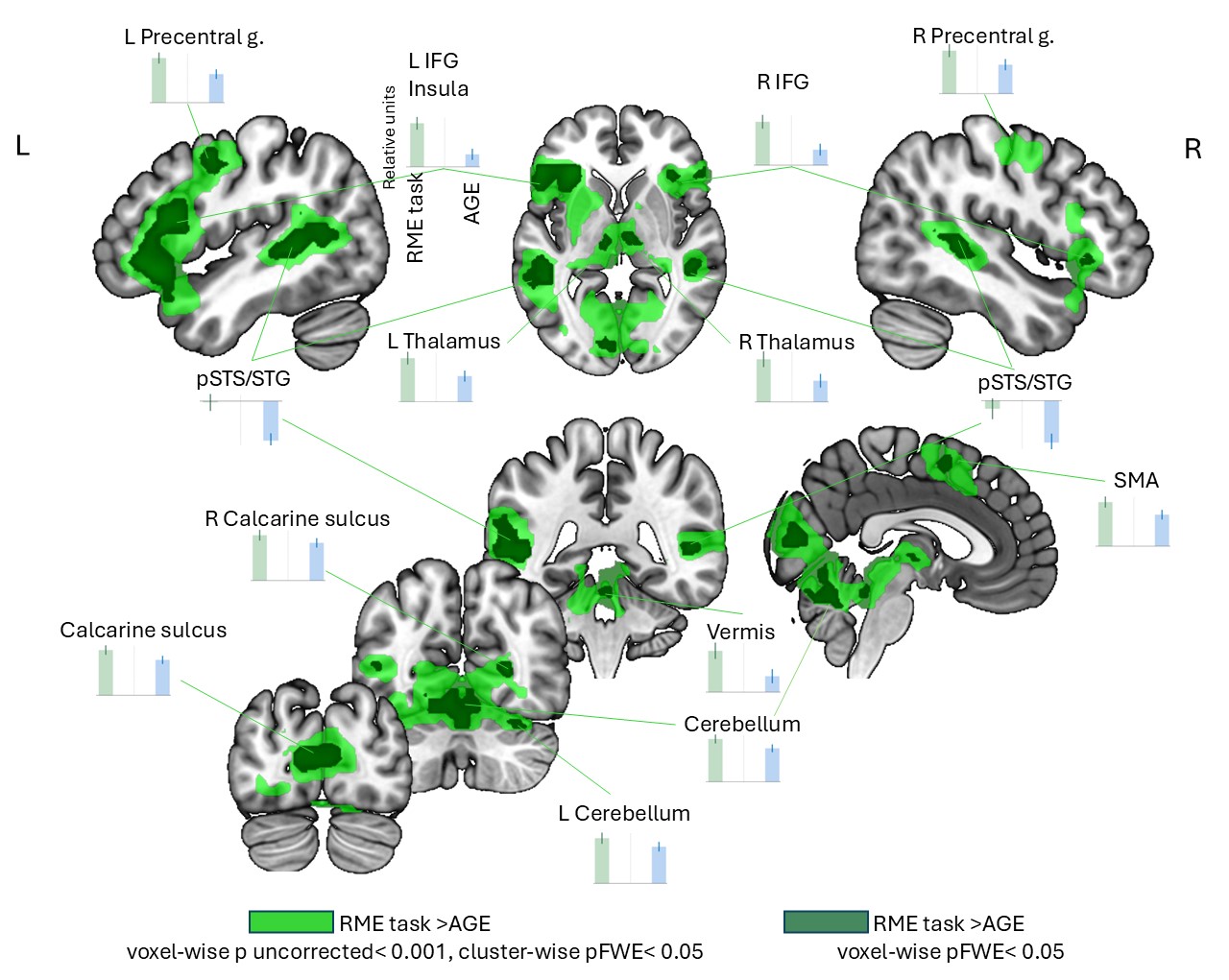


**Figure S1.** Clusters of increased BOLD signal during recognition of affective mental states from the eyes (RME task) compared to age identification (AGE). Plots show effect sizes for conditions of interest (vs. baseline) with 90% confidence intervals.

1. **FIR Analysis Within the Clusters of Significant BOLD Signal Changes Associated with the RME Task**

Second, brain regions showing increased BOLD signal during the RME task compared to the AGE condition were used as a mask, and FIR analysis was performed within this mask. This allowed the separation of clusters showing significant standard above-baseline activations and below-baseline BOLD signal changes. Table S2 and Figure S2 present the results of the FIR analysis. Remarkably, in Figure S2, clusters shown in green illustrate the same results of the standard RME task > AGE comparison as Figure S1.

**Table S2.** Regions identified by FIR analysis showing different types of BOLD signal changes relative to baseline within clusters defined in the standard analysis of BOLD signal changes during the RME task compared to the AGE control condition (voxel-wise FWE-corrected p < 0.05, cluster size threshold = 10). Each region is aligned with the corresponding subregions of the Brainnetome Atlas (Fan et al., 2016).

| **Anatomical localization** | **BNT subregion** | **Cluster** | **Peak** | | | | | |
| --- | --- | --- | --- | --- | --- | --- | --- | --- |
|  |  | **k** | **T** | **Z** | **p(FWE-corr)** | **x** | **y** | **z** |
| **RME task > AGE**  **Above-baseline BOLD signal during the RME task** | | | | | | | | |
| L IFG, Insula, Precentral g., Postcentral g., Temporal pole, Cuneus, Superior/middle/inferior occipital g., Fusiform g., Lingual g., Calcarine g., Cerebellum, Putamen, Caudate n., Thalamus, Hippocampus  R Temporal pole, Cuneus, Superior occipital g., Fusiform g., Lingual g., Calcarine g., Cerebellum, Caudate n., Thalamus, Hippocampus | L rostral lingual gyrus; L caudal cuneus gyrus; L caudal ventrolateral area 6; L medioventral area 37; R rostral cuneus gyrus; R rostral lingual gyrus; R caudal cuneus gyrus; L ventromedial parieto-occipital sulcus; L area 44 opercular; L medial superior occipital gyrus; L area 4 head-face; R area 44 dorsal; L area 6 caudal dorsolateral; L area 12/47 lateral; L area 37 lateroventral; L area 45 caudal; L caudal cuneus gyrus; L area 44 ventral; L area 45 rostral; R ventromedial parieto-occipital sulcus; L dorsolateral putamen; L area 38 lateral; L ventromedial putamen; L dorsal insula anterior; L inferior frontal junction; L lateral prefrontal thalamus; L caudal lingual gyrus; L inferior frontal sulcus; R lateral prefrontal thalamus; L inferior occipital gyrus; R dorsal caudate; L occipital thalamus; L posterior parietal thalamus; L middle occipital gyrus; L globus pallidus; L caudal hippocampus; R medial superior occipital gyrus; L area 12/47 orbital; R caudal hippocampus; L area 6 ventrolateral; L area 1/2/3 upper limb head-face; R posterior parietal thalamus; L ventral insula anterior; L occipital pole cortex; R caudal lingual gyrus | 5632 | 49.71 | >8 | <0.001 | -6 | -82 | 5 |
|  |  |  | 47.92 |  |  | 12 | -85 | 8 |
|  |  |  | 41.67 |  |  | -12 | -76 | -7 |
| L SMA | R area 8 medial; L area 8 medial; R area 6 medial; L area 6 medial; R area 32 posterior; R area 24 caudal dorsal | 548 | 25.14 | >8 | <0.001 | -3 | 11 | 50 |
| R IFG | R area 44 opercular; R area 12/47 lateral; R area 45 caudal; R dorsal insula anterior; R area 38 lateral; R area 45 rostral | 505 | 17.81 | >8 | <0.001 | 33 | 23 | -1 |
|  |  |  | 17.58 |  |  | 39 | 20 | 20 |
|  |  |  | 9.68 |  |  | 51 | 20 | -16 |
| R Precentral g/MFG | R area 6 caudal dorsolateral; R area 1/2/3 upper limb head-face; R area 4 upper limb | 257 | 16.52 | >8 | <0.001 | 39 | -1 | 53 |
| L STG | L rostroposterior STS; L caudoposterior STS | 119 | 10.59 | >8 | <0.001 | -48 | -43 | 5 |
|  |  |  | 5.51 |  |  | -39 | -61 | 2 |
| R STG | R rostroposterior STS | 21 | 6.36 | 6.35 | <0.001 | 51 | -37 | 5 |
| **RMET task > AGE**  **Below-baseline BOLD signal during the RME task** | | | | | | | | |
| L STG/MTG/AG | L dorsolateral area 37; L caudal area 22; L rostroventral area 39 (PGa) | 92 | 10.65 | >8 | <0.001 | -60 | -61 | 14 |
|  |  |  | 9.91 |  |  | -54 | -67 | 11 |
|  |  |  | 9.55 |  |  | -63 | -37 | 20 |
| R MTG/AG | R dorsolateral area 37; R rostroventral area 39 (PGa) | 52 | 10.13 | >8 | <0.001 | 66 | -46 | 17 |
|  |  |  | 9.85 |  |  | 63 | -55 | 14 |


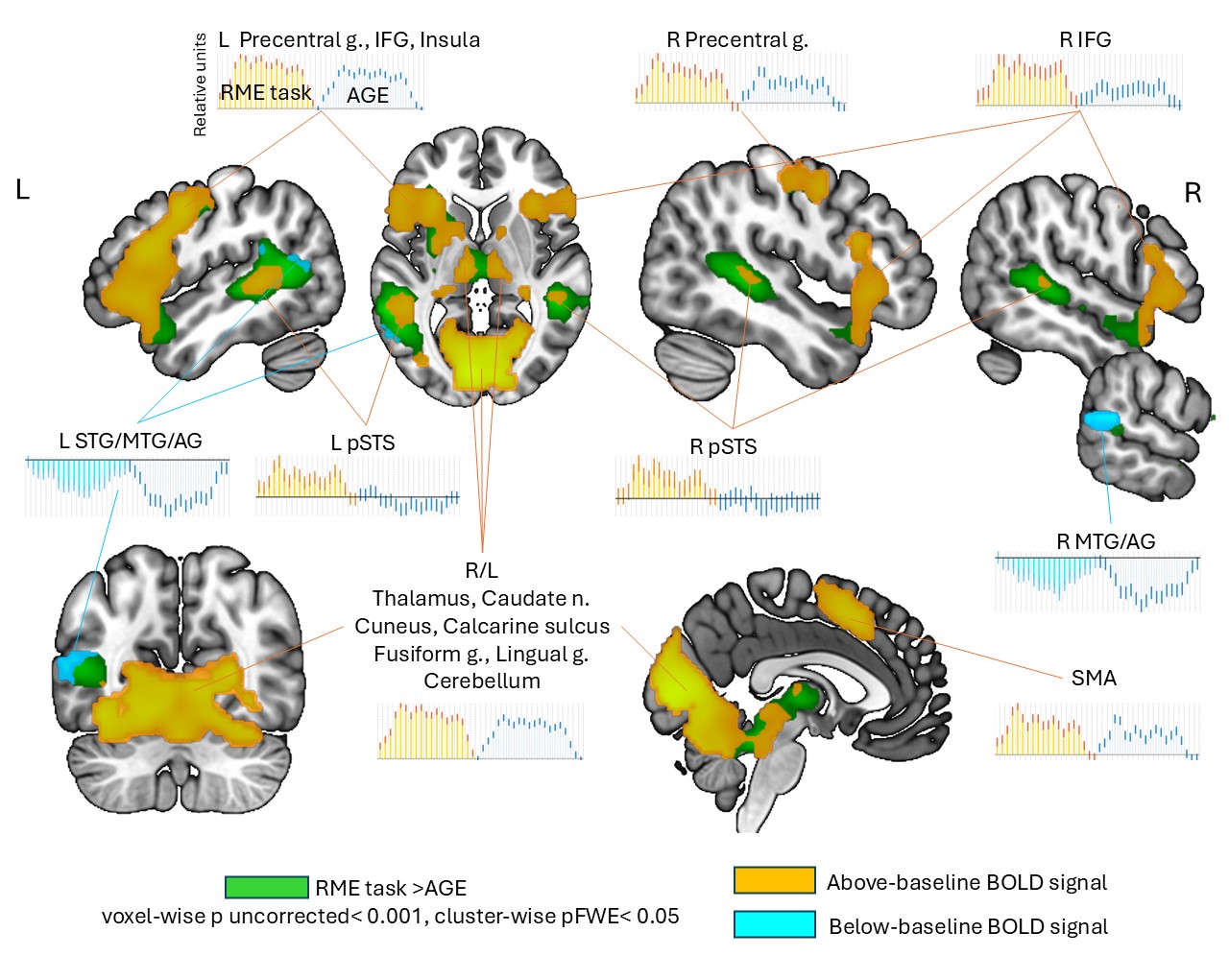


**Figure S2.** Clusters of increased BOLD signal during recognition of affective mental states from the eyes compared to age identification according to standard analysis are shown in green. Within these clusters, subregions exhibiting increased RME task > AGE BOLD signal reaction above baseline are shown in orange. Subregions showing increased RME task > AGE BOLD signal reaction but observed below baseline are shown in teal. Plots show effect sizes for regressors in the FIR models (vs. baseline) with 90% confidence intervals

1. **Defining ROIs for the TMFC Analysis**

Third, to create regions of interest (ROIs) for the task-modulated functional connectivity (TMFC) analysis, clusters showing significant above- and below-baseline BOLD signal changes according to the FIR analysis were further subdivided into finer areas by masking them with the Brainnetome Atlas parcellation. These ROIs included three groups: 1) located within the left and right TPJ-pSTS regions with below-baseline BOLD signal; 2) located within the left and right TPJ-pSTS regions with above-baseline activation; 3) located outside the left and right TPJ-pSTS regions. As the main focus of the present study was on determining the functional role of ToM network brain areas with BOLD signal changes below baseline during the RME task, Figure S3 illustrates ROIs within the TPJ-pSTS region, including those showing this type of BOLD signal changes.


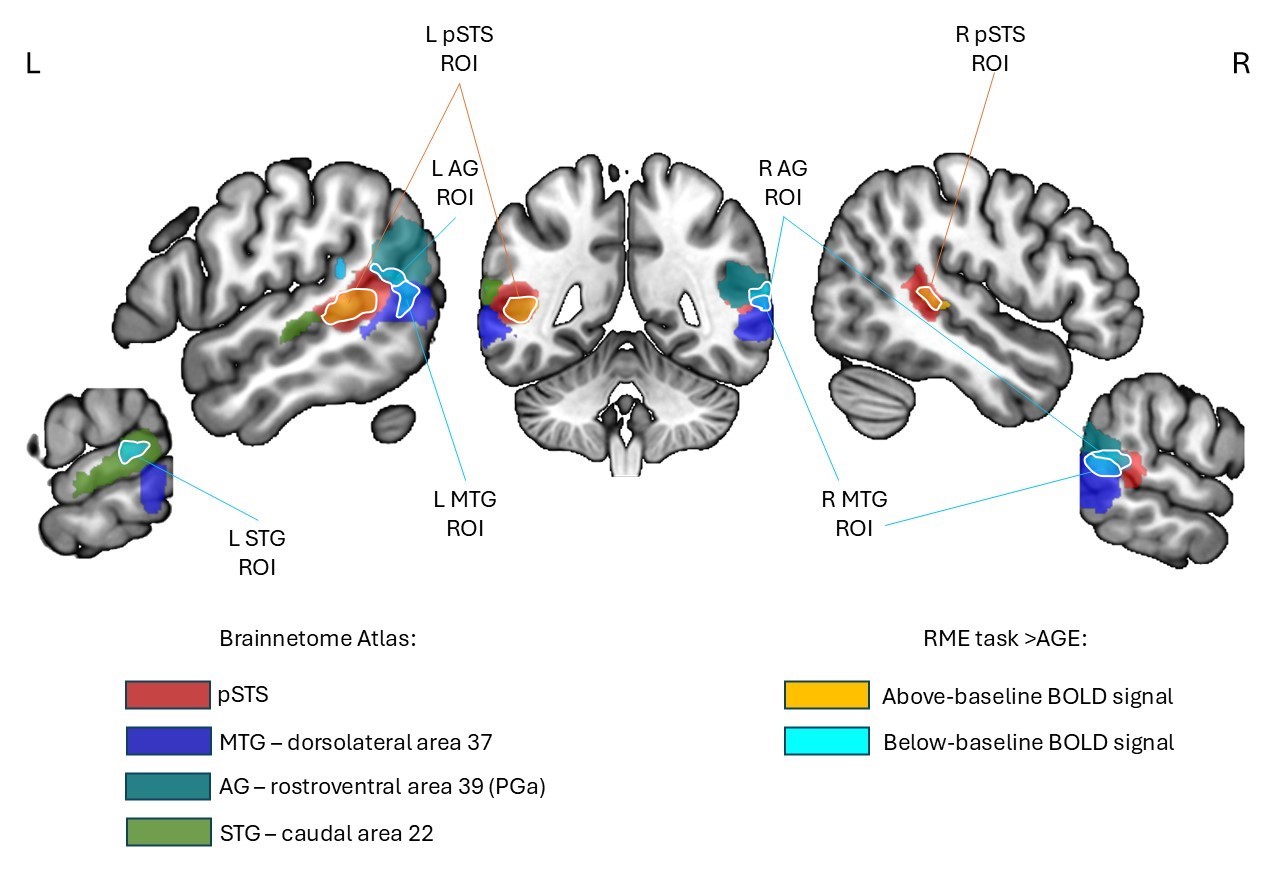


Figure S3. ROIs for the TMFC analysis located within the TPJ-pSTS region

1. **RME Task-induced TMFC Changes**

**Table S3.** RME task-induced increases of TMFC for ROIs showing above-baseline activation during the RME task (ROIs outside the TPJ-pSTS region) (voxel-wise uncorrected p < 0.001, cluster-wise FWE-corrected p < 0.05). Each region is aligned with the corresponding subregions of the Brainnetome Atlas (Fan et al., 2016).

| **BNT region** | **BNT subregion** | **Cluster** | | **Peak** | | | | | |
| --- | --- | --- | --- | --- | --- | --- | --- | --- | --- |
|  |  | **p(FWE-corr)** | **k** | **T** | **Z** | **p(unc)** | **x** | **y** | **z** |
| **TMFC increase for the ROI in the left STG BA 38** | | | | | | | | | |
| Right  Precentr g. /  MFG | Caudal ventrolateral area 6/  Inferior frontal junction | 0.036 | 86 | 4.4 | 4 | <0.001 | 36 | 5 | 41 |
| Right  SOG /  Angular g /  SMG | Lateral superior occipital g./  Rostrodorsal area 39(Hip3) /  Rostrodorsal area 40(PFt) | 0.002 | 172 | 4.3 | 4 |  | 30 | -55 | 44 |
| Left  SOG /  Angular g. /  SMG /  SPL | Lateral superior occipital g./  Rostrodorsal area 39(Hip3);  Caudal area 39 (PGp) /  Rostrodorsal area 40(PFt) /  Intraparietal area 7(hIP3);  Lateral area 5 | <0.001 | 278 | 4.9 | 4.5 |  | -36 | -55 | 47 |
| **TMFC increase for the ROI in the right dorsal agranular insula** | | | | | | | | | |
| Left  IFG | Caudal area 45;  Opercular area 44 | 0.05 | 74 | 4.16 | 3.9 |  | -42 | 26 | 2 |
| **TMFC increase for the ROI in the left IFG caudal area 45** | | | | | | | | | |
| Left  Precentral g. /  IFG | Caudal ventrolateral area 6 / Dorsal area 44 | 0.021 | 110 | 4.2 | 3.9 |  | -39 | 11 | 26 |
| **TMFC increase for the ROI in the right IFG caudal area 45** | | | | | | | | | |
| Left  IFG | Inferior frontal sulcus  Caudal area 45 | 0.019 | 106 | 4.6 | 4.2 |  | -42 | 23 | 17 |
| **TMFC increase for the ROI in the left IFG ventral area 44** | | | | | | | | | |
| Left  Precental g. /  IFG | Caudal ventrolateral BA6 /  Dorsal area 44 | 0.026 | 92 | 4.3 | 4 |  | -42 | 23 | 17 |
| **TMFC increase for the ROI in the left Orbital g.** **lateral area 12/47** | | | | | | | | | |
| Left  Precentral g./  IFG / | Caudal ventrolateral area 6 /  Dorsal area 44;  Inferior frontal sulcus/ | <0.001 | 194 | 5.1 | 4.6 |  | -42 | 5 | 29 |
| Right  Precentral g. /  IFG /  MFG | Caudal ventrolateral area 6 /  Dorsal area 44  Inferior frontal junction | <0.001 | 190 | 5.2 | 4.7 |  | 42 | 23 | 20 |
| Left  SOG /  Angular g /  SPL | Lateral superior occipital g. /  Rostrodorsal area 39(Hip3);  Caudal area 39(PGp) /  Intraparietal area 7(hIP3) | <0.001 | 218 | 5.1 | 4.7 |  | -27 | -61 | 41 |

**Table S4.** RME task-induced decreases of TMFC for ROIs showing greater BOLD responses during the RME task (voxel-wise uncorrected p < 0.001, cluster-wise FWE-corrected p < 0.05). Each region is aligned with the corresponding subregions of the Brainnetome Atlas (Fan et al., 2016).

| **BNT region** | **BNT subregion** | **Cluster** | | **Peak** | | | | | |
| --- | --- | --- | --- | --- | --- | --- | --- | --- | --- |
|  |  | **p(FWE-corr)** | **k** | **T** | **Z** | **p(unc)** | **x** | **y** | **z** |
| **TMFC decrease for the ROI in the right pSTS** | | | | | | | | | |
| R. SMG | Caudal area 40 (PFm);  Rostroventral area 40(PFop) | 0.043 | 83 | 4.25 | 3.95 | <0.001 | 60 | -43 | 32 |
| **TMFC decrease for the ROI in the left caudal lingual gyrus of the cuneus** | | | | | | | | | |
| Right  Precentral g | Caudal ventrolateral area 6 | 0.045 | 82 | 4.8 | 4.4 |  | 57 | 11 | 23 |
| **TMFC decrease for the** **ROI** **in the left** **globus pallidus** | | | | | | | | | |
| Left  SFG /  Cingulate g. | Medial area 10 /  Subgenual area 32 | 0.032 | 88 | 4.5 | 4.2 |  | 0 | 50 | -1 |
| Left  MFG | Dorsal area 9/46;  Ventral area 9/46;  Area 46 | 0.004 | 149 | 4.7 | 4.3 |  | -30 | 44 | 26 |
| Left  Cingulate g /  Right  Cingulate g. | Pregenual area 32 /  Caudodorsal area 24 | 0.035 | 86 | 4.6 | 4.3 |  | 0 | 26 | 29 |
| Left  Precuneus | Medial area 7(PEp);  Area 31 (Lc1) | 0.009 | 122 | 4.6 | 4.3 |  | -6 | -55 | 50 |
| **TMFC decrease for the ROI in the** **left medial area 8 of the SFG** | | | | | | | | | |
| Left  Cingulate g. /  SFG | Subgenual area 32;  Medial area 10 | <0.001 | 78 | 4.4 | 4.1 | <0.001 | -3 | 53 | -1 |
| **TMFC decrease for the ROI** **in** **the left medial Pre-frontal thalamus** | | | | | | | | | |
| Left/Right  SFG | Medial area 10 | 0.025 | 89 | 4.1 | 3.8 |  | -3 | 56 | 5 |
| Left  Precuneus | Area 31 (Lc1) | 0.035 | 81 | 4.1 | 3.8 |  | -6 | -49 | 26 |
| **TMFC decrease for the ROI** **in** **the left dorsal agranular insula** | | | | | | | | | |
| Left  Cingulate g  Left/Right  SFG | Subgenual area 32 /  Medial area 14 | 0.01 | 109 | 4.8 | 4.2 |  | -3 | 50 | -1 |
| Left/Right  Cingulate g. /  Left  Cingulate g. /  Precuneus | Сaudal area 23 /  Dorsal area 23 /  Area 31 (Lc1) | <0.001 | 417 | 5.5 | 4.9 |  | 9 | -22 | 44 |
| **TMFC decrease for the ROI in** **the** **left dorsal area 44 of the IFG** | | | | | | | | | |
| Left/Right  Cingulate g. /  Precuneus  Left  Precuneus /  Paracentr. l. /  Hippocamp. /  Fusiform g. | Dorsal area 23 /  Ventral area 23 /  Caudal area 23 /  Area 31 (Lc1) /  Dors. parietooccip. s.(PEr) /  Area1/2/3 (lower limb region) /  Caudal hippocampus /  Rostroventral area 20 | <0.001 | 716 | 5.1 | 4.7 |  | -6 | -52 | 11 |
| Left / Right  Cingulate g. /  SFG g . | Subgenual area 32 /  Medial area 14 | 0.008 | 130 | 4.6 | 4.3 |  | -3 | 53 | -4 |
| Left  MFG /  SFG | Ventrolateral area 8 /  Dorsolateral area 8 | 0.018 | 107 | 4.5 | 4.2 |  | -21 | 26 | 56 |
| Left  Angular g. /  SMG | Rostroventral area 39(PGa);  Rostrodorsal area 39(Hip3);  Caudal area 40(PFm) | 0.001 | 198 | 4.3 | 4 |  | -54 | -40 | 50 |
| **TMFC decrease for the ROI** **in** **the** **left caudal area 45 of the IFG** | | | | | | | | | |
| Left/Right  Precuneus /  Cingulate g.  Left  Precuneus | Area 31 (Lc1);  Caudal area 23;  Dorsal area 23;  Ventral area 23 /  Dors. parietooccip. s.(PEr) | <0.001 | 636 | 5.6 | 5 |  | -6 | -49 | 26 |
| Left  SFG  Cingulate g. /  MFG /  Right  SFG /  Cingulate g. | Medial area 10;  Dorsolateral area 8;  Lateral area 9 /  Subgenual area 32;  Pregenual area 32;  Dorsal area 9/46;  Area 46;  Ventrolateral area 8;  Inferior frontal junction /  Medial area 10;  Medial area 14;  Subgenual area 32 | <0.001 | 1013 | 5.5 | 5 |  | -15 | 32 | 53 |
| Left SMG | Caudal area 40(PFm) | <0.001 | 330 | 5.4 | 4.8 |  | -51 | -58 | 32 |
| **TMFC decrease for the ROI in the** **left rostral area 45 of the IFG** | | | | | | | | | |
| Left / Right  Cingulate g. /  Left  SFG / | Subgenual area 32;  Medial area 14/  Medial area 10 | <0.001 | 212 | 4.7 | 4.3 |  | 0 | 50 | -1 |
| Left  Cingulate g.  Left / Right  Precuneus / | Dorsal area 23;  Ventral area 23;  Caudal area 23;    Dors. parietooccip. s.(PEr);  Area 31 (Lc1) | <0.001 | 341 | 4.8 | 4.4 |  | -6 | -55 | 20 |
| Left  Angular g./  SMG | Rostroventral area 39(PGa) /  Caudal area 40(PFm) | <0.001 | 205 | 4.9 | 4.5 |  | -51 | -58 | 32 |
| **TMFC decrease for the ROI** **in** **the** **left opercular area 44 of the IFG** | | | | | | | | | |
| Left/ Right  SFG /  MFG  Cingulate g. | Medial area 10 /  Medial area 14 /  Dorsal area 9/46;  Area 46 /  Pregenual area 32;  Subgenual area 32 | <0.001 | 645 | 6.3 | 5.5 |  | -3 | 50 | -1 |
| Left/ Right  Precuneus /  Cingulate g.  Right  Paracentr. l. / | Area 31 (Lc1);  Dors. parietooccip. s.(PEr)/  Caudal area 23;  Dorsal area 23;  Ventral area 23 /  Area1/2/3 (lower limb region) | <0.001 | 988 | 6.1 | 5.4 |  | -9 | -46 | 29 |
| **TMFC decrease for the ROI in the** **right opercular area 44 of the IFG** | | | | | | | | | |
| Left  Cingulate g. | Caudal area 23 | 0.041 | 79 | 4.2 | 3.9 |  | -6 | -28 | 47 |
| **TMFC decrease for the ROI** **in the** **left** **ventral area 44 of the IFG** | | | | | | | | | |
| Left/Right  Cingulate g.  SFG | Subgenual area 32;  Pregenual area 32 /  Medial area 14 /  Medial area 10 | <0.001 | 225 | 5 | 4.5 |  | 0 | 44 | 11 |
| Left/Right  Precuneus /  Cingulate g. | Area 31 (Lc1) /  Dorsal area 23;  Caudal area 23 | <0.001 | 462 | 5.3 | 4.8 |  | -3 | -19 | 41 |
| Left  Angular g. /  SMG | Rostroventral area 39(PGa) /  Caudal area 40(PFm) | 0.025 | 93 | 4.3 | 4.6 |  | -51 | -58 | 32 |
| **TMFC decrease for the ROI in the** **left** **area 12/47 of the orbital gyrus** | | | | | | | | | |
| Left/Right  Cingulate g.  SFG | Subgenual area 32;  Pregenual area 32 /  Medial area 14 /  Medial area 10 | <0.001 | 300 | 4.9 | 4.4 |  | -3 | 53 | -1 |
| Left  Cingulate g. /  Precuneus | Dorsal area 23 /  Area 31 (Lc1) | 0.003 | 143 | 4.2 | 3.9 |  | -9 | -49 | 29 |
| Left  Angular g. /  SMG | Rostroventral area 39(PGa) /  Caudal area 40(PFm) | 0.016 | 101 | 4.5 | 4.2 |  | -51 | -58 | 32 |
| Left/Right  Cingulate g. | Caudal area 23 | 0.016 | 101 | 4.1 | 3.9 |  | -3 | -19 | 41 |
| **TMFC decrease for the ROI in the right area 12/47 of the orbital gyrus** | | | | | | | | | |
| Left/Right  Cingulate g. | Caudal area 23 | 0.017 | 99 | 4.5 | 4.1 |  | 9 | -25 | 44 |

1. **Response Time and RME Task Accuracy Effects on BOLD Signal and TMFC Changes, Associated with the RME Task**

To address the reviewer’s concern regarding the influence of differences in response time (RT) between experimental conditions and individual accuracy of RME task performance on the observed BOLD signal and TMFC changes between the RME task and the AGE (age identification) control condition, we performed additional analyses at the second level. Subject- and condition-specific behavioral variables were added as covariates in separate models. Prior to inclusion, RT and accuracy data (expressed in percent) were mean-centered and z-transformed to ensure normality of their distribution.

To assess the impact of RT on the observed BOLD signal changes, we re-estimated the one-sample *t*-test model for the RME task > AGE contrast while including the RT difference between the RME task and AGE condition as a covariate of no interest. This analysis revealed significant BOLD signal changes highly consistent with the main results reported in the text of the manuscript (see Table S5 and Figure S4). An analogous re-analysis, including RME task accuracy as a covariate of no interest in the same model, produced similar results, replicating the initial findings (see Table S5 and Figure S4).

**Table S5.** Clusters showing relative BOLD signal increases associated with the RME task (RME task > AGE), controlling for RT and RME task accuracy, included as covariates of no interest at the second-level (voxel-wise uncorrected p < 0.001, cluster-wise FWE-corrected p < 0.05, cluster size threshold = 10).

| **Anatomical localization** | **Cluster** | | | | | **Peak** | | | | | | | |
| --- | --- | --- | --- | --- | --- | --- | --- | --- | --- | --- | --- | --- | --- |
|  | **p(FWE-corr)** | | | **k** | | **T** | | **Z** | | **p(unc)** | **x** | **y** | **z** |
| **RME task >AGE (RT difference between the RME task and AGE condition as a covariate of no interest** | | | | | | | | | | | | | |
| L IFG, Insula, Precentral g., Postcentral g., STG, MTG, Temporal pole, Superior/middle/inferior occipital g., Fusiform g., Lingual g., Calcarine g., Cerebellum, Putamen, Caudate n., Thalamus, Hippocampus  R STG, MTG, Temporal pole, Cuneus, Superior occipital g., Fusiform g., Lingual g., Calcarine g., Cerebellum, Caudate n., Thalamus, Hippocampus | | <0.001 | 7450 | | 7.93 | | 6.50 | | <0.001 | | -39 | -7 | 38 |
|  |  |  |  |  | 7.75 | | 6.40 | |  |  | -54 | 20 | 17 |
|  |  |  |  |  | 7.68 | | 6.35 | |  |  | -33 | 26 | -1 |
| R IFG | | <0.001 | 622 | | 6.22 | | 5.42 | | <0.001 | | 48 | 23 | -4 |
|  |  |  |  |  | 6.20 | | 5.41 | |  |  | 33 | 26 | -1 |
|  |  |  |  |  | 5.66 | | 5.03 | |  |  | 57 | 26 | 5 |
| L SMA | | <0.001 | 621 | | 6.20 | | 5.41 | | <0.001 | | -6 | 17 | 44 |
|  |  |  |  |  | 5.90 | | 5.20 | |  |  | 0 | 8 | 59 |
|  |  |  |  |  | 5.03 | | 4.56 | |  |  | 9 | 14 | 41 |
| R Precentral g./MFG | | <0.001 | 270 | | 5.41 | | 4.85 | | <0.001 | | 42 | -1 | 47 |
|  |  |  |  |  | 5.03 | | 4.56 | |  |  | 42 | -10 | 53 |
|  |  |  |  |  | 4.18 | | 3.89 | |  |  | 33 | -10 | 62 |
| **RME task >AGE (RME task accuracy in percent as a covariate of no interest)** | | | | | | | | | | | | | |
| L IFG, Insula, Precentral g., Postcentral g., STG, MTG, Temporal pole, Superior/middle/inferior occipital g., Fusiform g., Lingual g., Calcarine g., Cerebellum, Putamen, Caudate n., Thalamus, Hippocampus  R STG, MTG, Temporal pole, Cuneus, Superior occipital g., Fusiform g., Lingual g., Calcarine g., Cerebellum, Caudate n., Thalamus, Hippocampus | | <0.001 | 7110 | | 7.53 | | 6.26 | | <0.001 | | -54 | 20 | 17 |
|  |  |  |  |  | 7.30 | | 6.13 | |  |  | -57 | -46 | 11 |
|  |  |  |  |  | 7.27 | | 6.11 | |  |  | -39 | -7 | 41 |
| R IFG | | <0.001 | 589 | | 6.06 | | 5.31 | | <0.001 | | 48 | 26 | -4 |
|  |  |  |  |  | 5.78 | | 5.11 | |  |  | 36 | 26 | -1 |
|  |  |  |  |  | 5.48 | | 4.90 | |  |  | 57 | 26 | 8 |
| L SMA | | <0.001 | 586 | | 5.88 | | 5.19 | | <0.001 | | -6 | 14 | 44 |
|  |  |  |  |  | 5.42 | | 5.18 | |  |  | 0 | 8 | 59 |
|  |  |  |  |  | 5.41 | | 4.85 | |  |  | 9 | 2 | 50 |
| R Precentral g./MFG | | <0.001 | 275 | | 5.30 | | 4.77 | | <0.001 | | 45 | -1 | 47 |
|  |  |  |  |  | 5.25 | | 4.73 | |  |  | 42 | -10 | 53 |
|  |  |  |  |  | 4.18 | | 3.89 | |  |  | 33 | -10 | 62 |

In addition, the parametric effect of RT on the BOLD signal difference (RME task > AGE) was assessed within the same model showing a positive correlation (see Table S6 and Figure S4). However, the observed effect only partially overlaps with the original findings of BOLD signal difference and is not located in the brain region of the main focus of the current study – the TPJ-pSTS regions.

**Table S6.** Clusters showing a positive correlation between RT (difference between the RME task and AGE condition) and BOLD signal increases associated with the RME task (RME task > AGE) (voxel-wise uncorrected p < 0.001, cluster-wise FWE-corrected p < 0.05, cluster size threshold = 10).

| **Anatomical localization** | **Cluster** | | **Peak** | | | | | |
| --- | --- | --- | --- | --- | --- | --- | --- | --- |
|  | **p(FWE-corr)** | **k** | **T** | **Z** | **p(unc)** | **x** | **y** | **z** |
| **Positive correlation between RT and the BOLD signal difference (RME task >AGE)** | | | | | | | | |
| Left middle and inferior occipital gyri, medial superior occipital gyrus, fusiform gyrus, superior parietal lobule (intraparietal area 7(hIP3) and postcentral area 7), angular gyrus (caudal area 39(PGp), precuneus | <0.001 | 865 | 6,29 | 5,47 | <0.001 | -24 | -61 | 53 |
|  |  |  | 6,09 | 5,33 |  | -24 | -73 | 32 |
|  |  |  | 5,36 | 4,81 |  | -27 | -52 | 38 |
| Left dorsal anterior insula, superior temporal gyrus (BA38), orbital gyrus (lateral area 12/47) | 0.003 | 181 | 4,88 | 4,45 |  | -30 | 20 | 2 |
|  |  |  | 4,46 | 4,12 |  | -42 | 17 | -16 |
|  |  |  | 4,05 | 3,79 |  | -45 | 17 | 11 |
| Bilateral rostral and caudal cuneus gyrus,  Right occipital polar cortex, rostral lingual gyrus, middle occipital gyrus | <0.001 | 415 | 4,95 | 4,50 |  | 39 | -85 | 2 |
|  |  |  | 4,72 | 4,33 |  | -3 | -97 | 20 |
|  |  |  | 4,41 | 4,08 |  | 3 | -88 | 2 |
| Left middle frontal gyrus (inferior frontal junction), precentral gyrus (caudal ventrolateral area 6), inferior frontal gyrus (dorsal BA 44), precentral gyrus (caudal dorsolateral area 6) | <0.001 | 475 | 5,95 | 5,23 |  | -42 | -1 | 29 |
|  |  |  | 5,53 | 4,93 |  | -42 | 17 | 32 |
|  |  |  | 5,02 | 4,56 |  | -48 | 11 | 38 |
| Bilateral superior frontal gyrus, including medial BA8 and BA9 | <0.001 | 290 | 6,07 | 5,32 |  | -3 | 29 | 41 |
|  |  |  | 5,12 | 4,63 |  | -9 | 11 | 53 |
|  |  |  | 4,58 | 4,22 |  | 3 | 17 | 50 |

**
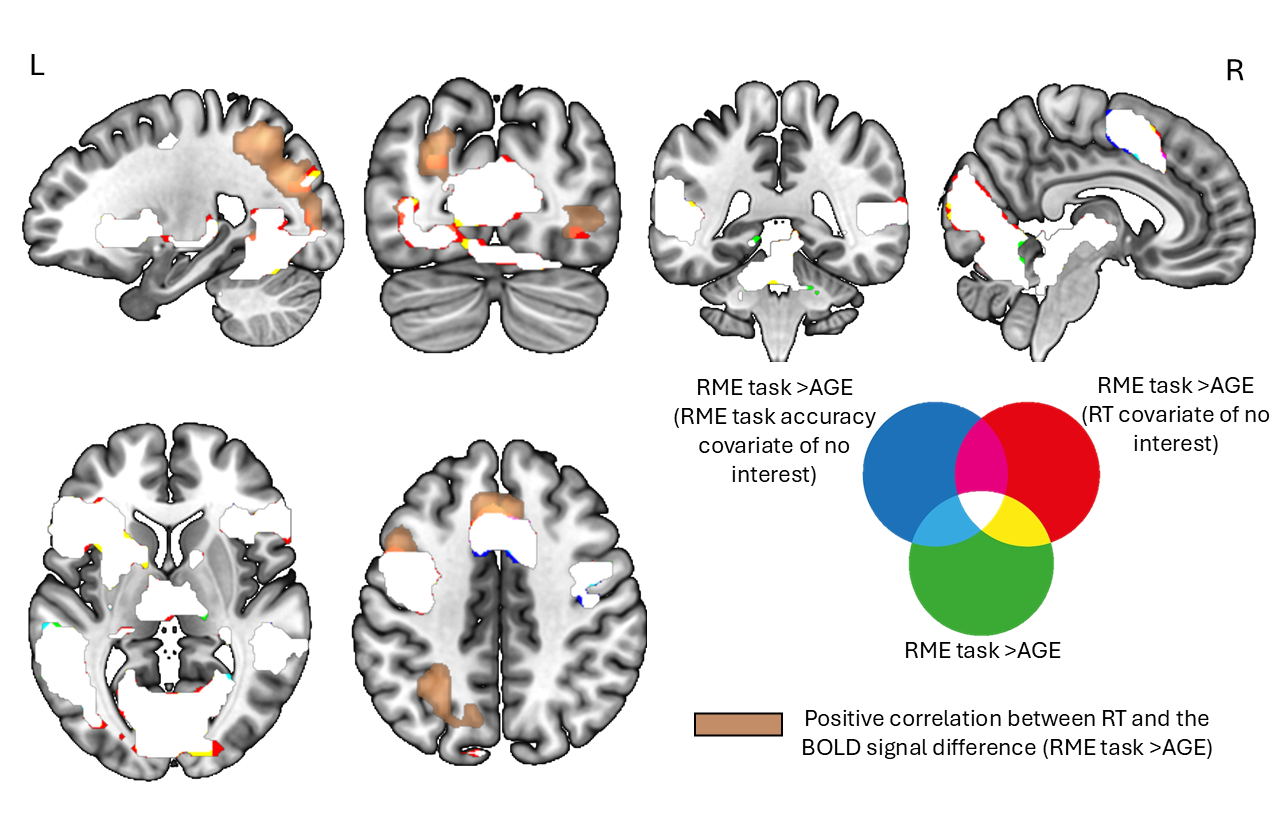
**

**Figure S4.** Overlap between clusters of increased BOLD signal associated with the RME task (vs AGE) obtained in the original analysis and in additional analyses including RT and RME task accuracy as covariates of no interest at the second level. The figure also illustrates the limited overlap between these clusters and those showing a positive correlation between RT and BOLD signal increases (RME task > AGE) (voxel-wise uncorrected p < 0.001, cluster-wise FWE-corrected p < 0.05).

We repeated this procedure for the TMFC analysis focusing on ROIs within the TPJ-pSTS region. The inclusion of RT and RME task accuracy covariates did not alter the original results: all effects remained unchanged, with only minor differences in the corresponding statistical parameters and cluster extent (see Table S7). As the anatomical localization of the results was consistent with the initial findings, Table S7 reports only broader BNT regions, for BNT subregions, see Table 2 and Table 3.

**Table S7.** RME task-induced increases in TMFC, controlling for RT and RME task accuracy (included as covariates of no interest at the second-level), for ROIs within the TPJ-pSTS region showing greater BOLD responses during the RME task, including ROIs with both above-baseline activations and responses remaining below baseline (voxel-wise uncorrected p < 0.001, cluster-wise FWE-corrected p < 0.05).

| **BNT region** | **Cluster p(FWE-corr)** | **k** | **T** | **Z** | **p(unc)** | **x** | **y** | **z** |
| --- | --- | --- | --- | --- | --- | --- | --- | --- |
| **RT difference between the RME task and AGE condition as a covariate of no interest** | | | | | | | | |
| **TMFC increase for ROI in left MTG (area 37)** | | | | | | | | |
| Right Occipital g. / SOG / AG / Fusiform g. / SMG / SPL | <0.001 | 1145 | 6.73 | 5.76 | <0.001 | 30 | -61 | 38 |
| Left Occipital g. / SOG / Fusiform g. / AG / SMG / SPL / Cuneus | <0.001 | 994 | 5.11 | 4.62 | <0.001 | -36 | -55 | -13 |
| Right Precentral g. / IFG / MFG | <0.001 | 267 | 4.34 | 4.02 | <0.001 | 42 | 23 | 26 |
| Left Precentral g. / IFG / MFG | <0.001 | 237 | 4.28 | 3.97 | <0.001 | -48 | 32 | 29 |
| Bilateral SFG | 0.002 | 173 | 5.83 | 5.15 | <0.001 | 6 | 20 | 50 |
| **TMFC increase for ROI in left AG (area 39)** | | | | | | | | |
| Right Occipital g. / Fusiform g. | 0.048 | 79 | 4.10 | 3.90 | <0.001 | 45 | -58 | -7 |
| Left Occipital g. / Fusiform g. | 0.020 | 103 | 4.09 | 3.82 | <0.001 | -42 | -73 | -1 |
| Right Occipital g. / SOG / AG / SMG / SPL | <0.001 | 594 | 6.12 | 5.35 | <0.001 | 30 | -58 | 35 |
| Left IFG / MFG | 0.010 | 123 | 4.80 | 4.39 | <0.001 | -48 | 32 | 32 |
| Right IFG / MFG | 0.002 | 176 | 4.62 | 4.25 | <0.001 | 48 | 32 | 26 |
| Left SOG / AG / SPL | <0.001 | 306 | 5.16 | 4.66 | <0.001 | -30 | -64 | 44 |
| **TMFC increase for ROI in left STG (area 22)** | | | | | | | | |
| Right AG / SOG | 0.010 | 128 | 4.34 | 4.02 | <0.001 | 36 | -52 | 44 |
| Left AG / SPL | 0.044 | 84 | 4.06 | 3.79 | <0.001 | -33 | -49 | 44 |
| **TMFC increase for ROI in right MTG (area 37)** | | | | | | | | |
| Right AG / SMG / SOG | <0.001 | 225 | 4.89 | 4.45 | <0.001 | 33 | -58 | 41 |
| **TMFC increase for ROI in right AG (area 39)** | | | | | | | | |
| Right AG / SMG / SOG | <0.001 | 249 | 4.59 | 4.22 | <0.001 | 30 | -58 | 38 |
| **TMFC increase for ROI in left pSTS** | | | | | | | | |
| Left Fusiform g. | 0.010 | 124 | 5.37 | 4.82 | <0.001 | -36 | -55 | -13 |
| Left Precentral g. / IFG | 0.005 | 145 | 4.88 | 4.45 | <0.001 | -42 | -1 | 32 |
| Right SOG | 0.007 | 132 | 4.76 | 4.36 | <0.001 | 33 | -70 | 26 |
| **TMFC increase for ROI in right pSTS** | | | | | | | | |
| Left SOG / AG | 0.045 | 82 | 4.35 | 4.03 | <0.001 | -30 | -58 | 38 |
| **RME task accuracy (in percent) as a covariate of no interest** | | | | | | | | |
| **TMFC increase for ROI in left MTG (area 37)** | | | | | | | | |
| Right Occipital g. / SOG / AG / Fusiform g. / SMG / SPL | <0.001 | 1169 | 6.68 | 5.73 | <0.001 | 30 | -61 | 38 |
| Left Occipital g. / SOG / Fusiform g. / AG / SMG / SPL / Cuneus | <0.001 | 1059 | 5.28 | 4.75 | <0.001 | -39 | -76 | 2 |
| Right Precentral g. / IFG / MFG | <0.001 | 281 | 4.39 | 4.06 | <0.001 | 48 | 32 | 26 |
| Left Precentral g. / IFG / MFG | <0.001 | 275 | 4.27 | 3.97 | <0.001 | -33 | 11 | 32 |
| Bilateral SFG | 0.002 | 174 | 5.82 | 5.14 | <0.001 | 6 | 20 | 50 |
| **TMFC increase for ROI in left AG (area 39)** | | | | | | | | |
| Right Occipital g. / Fusiform g. | 0.048 | 79 | 4.10 | 3.90 | <0.001 | 45 | -58 | -7 |
| Left Occipital g. / Fusiform g. | 0.019 | 103 | 4.10 | 3.80 | <0.001 | -42 | -73 | -1 |
| Right Occipital g. / SOG / AG / SMG / SPL | <0.001 | 602 | 6.12 | 5.36 | <0.001 | 30 | -58 | 35 |
| Left IFG / MFG | 0.008 | 129 | 4.67 | 4.28 | <0.001 | -48 | 32 | 32 |
| Right IFG / MFG | 0.001 | 180 | 4.67 | 4.28 | <0.001 | 48 | 32 | 26 |
| Left SOG / AG / SPL | <0.001 | 305 | 5.22 | 4.70 | <0.001 | -30 | -64 | 44 |
| **TMFC increase for ROI in left STG (area 22)** | | | | | | | | |
| Right AG / SOG | 0.018 | 109 | 4.30 | 3.90 | <0.001 | 36 | -52 | 44 |
| Left AG / SPL | 0.042 | 106 | 4.07 | 3.80 | <0.001 | -33 | -49 | 44 |
| **TMFC increase for ROI in right MTG (area 37)** | | | | | | | | |
| Right AG / SMG / SOG | <0.001 | 227 | 4.83 | 4.41 | <0.001 | 33 | -58 | 41 |
| **TMFC increase for ROI in right AG (area 39)** | | | | | | | | |
| Right AG / SMG / SOG | <0.001 | 245 | 4.52 | 4.17 | <0.001 | 30 | -58 | 38 |
| **TMFC increase for ROI in left pSTS** | | | | | | | | |
| Left Fusiform g. | 0.0081 | 129 | 5.35 | 4.80 | <0.001 | -36 | -55 | -13 |
| Left Precentral g. / IFG | 0.0046 | 146 | 4.87 | 4.44 | <0.001 | -42 | -1 | 32 |
| Right SOG | 0.0083 | 128 | 4.76 | 4.35 | <0.001 | 33 | -70 | 26 |
| **TMFC increase for ROI in right pSTS** | | | | | | | | |
| Left SOG / AG | 0.030 | 91 | 4.49 | 4.14 | <0.001 | -30 | -58 | 38 |

Overall, these results indicate that the reported BOLD signal and TMFC effects are robust and not driven by differences in RT or task accuracy. Importantly, although RT showed a partial positive association with BOLD signal changes, this effect was spatially distinct from the main findings and did not involve the TPJ-pSTS regions. This supports the interpretation that the observed effects reported in the main manuscript are independent of between-condition differences in task difficulty and individual differences in the ability to recognize affective states.
